# Microvascular autophagy and caspase-3 activation are central regulators of renal fibrosis after ischemia-reperfusion

**DOI:** 10.1172/jci.insight.201945

**Published:** 2026-07-08

**Authors:** Hyunyun Kim, Francis Migneault, Shanshan Lan, Imane Kaci, Julie Turgeon, Annie Karakeussian Rimbaud, Martin Dupont, Shijie Qi, Mélanie Dieudé, Marie-Josée Hébert

**Affiliations:** 1 Département de Médecine, Université de Montréal, Montréal, Quebec, Canada.; 2 Centre de Recherche, Centre Hospitalier de l’Université de Montréal (CRCHUM), Montréal, Quebec, Canada.; 3 Canadian Donation and Transplantation Research Program (CDTRP), University of Alberta, Edmonton, Alberta, Canada.; 4 Département de Microbiologie, Infectiologie et Immunologie, Faculté de Médecine, Université de Montréal, Montréal, Quebec, Canada.; 5 Medical Affairs and Innovation, Héma-Québec, Québec, Quebec, Canada.

**Keywords:** Nephrology, Vascular biology, Apoptosis, Autophagy, Fibrosis

## Abstract

Ischemia-reperfusion injury (IRI) is a common cause of acute kidney injury (AKI) leading to renal fibrosis. Here, we investigate the kinetics of autophagy, apoptosis, and necroptosis activation in tubular epithelial cells (TECs) and peritubular capillaries (PTCs) after renal IRI, and their relative contributions to renal fibrogenesis. IRI with renal artery clamping in GFP-LC3 transgenic mice induced a predominant and sustained necroptotic response in TECs, while apoptosis and autophagy played minor roles. PTCs showed early and persistent activation of apoptosis, brief necroptosis induction, and increased autophagy at a distance from IRI. Disruption of the autophagic process with chloroquine (CHQ) injections in association with renal IRI did not modulate tubular death but enhanced PTC apoptosis and increased microvascular rarefaction and fibrosis. Apoptosis-deficient GFP-LC3*/Caspase-3^–/–^* mice exposed to renal IRI showed enhanced PTC autophagy, reduced PTC rarefaction, and inhibition of renal fibrosis, in spite of increased necroptosis in TECs. Inhibition of both autophagy with CHQ and apoptosis in GFP-LC3*/Caspase-3^–/–^* mice led to a marked switch toward necroptosis in PTCs. This was associated with aggravated microvascular rarefaction, increased leukocyte infiltration, and enhanced renal fibrosis. These findings establish a predominant role for PTC autophagy and caspase-3–dependent apoptosis in the development of renal fibrosis after IRI.

## Graphical abstract

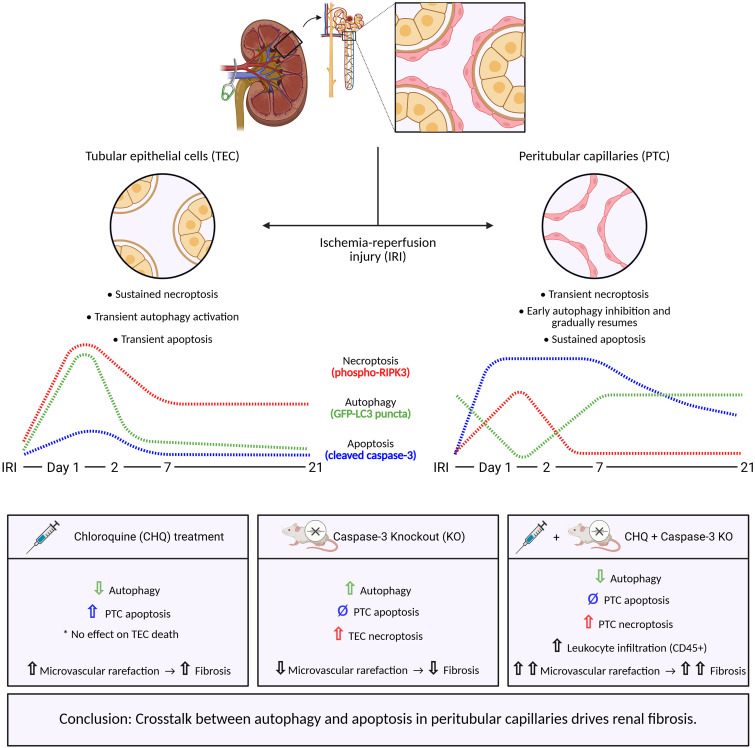

## Introduction

Ischemia-reperfusion injury (IRI) is a major cause of acute kidney injury (AKI), which can progress to chronic kidney disease (CKD), characterized by renal fibrosis and ultimately renal failure (1). Renal fibrosis, an early predictor of end-stage renal failure, is associated with excessive and persistent accumulation of extracellular matrix (ECM) components, including collagens, fibronectin, and laminin, within the interstitial compartment (2). Persistent activation of renal fibroblasts and myofibroblast differentiation, together with ongoing inflammation and capillary rarefaction fuel changes in tissue architecture leading to permanent renal dysfunction (3, 4).

IRI-induced death of renal parenchymal cells leading to production of damage-associated molecular patterns (DAMPs), inflammation, and microvascular rarefaction all of which are considered central to renal fibrogenesis (4). Many types of regulated cell death programs have been identified within renal cells over the past decades, with apoptosis and necroptosis being the best characterized (5–7). Apoptosis is initiated through extrinsic or intrinsic signals that ultimately center on activation of effector caspases, such as caspase-3 or -7. Necroptosis, a proinflammatory mode of programmed cell death associated with release of DAMPs, is mediated by the phosphorylation of receptor-interacting kinase-3 (RIPK3) which in turn phosphorylates downstream targets. Autophagy, a stress response and survival mechanism based on the degradation of unwanted or damaged cellular constituents, can prevent activation of death regulated pathways (8). Apoptosis, necroptosis, and autophagy have been identified within tubular epithelial cells (TECs) after renal IRI, but their relative importance and contribution to renal fibrogenesis remain to be fully characterized. For example, IRI-induced AKI in renal proximal tubule–specific *Atg5*-KO mice results in worsened renal function, enhanced renal tubular damage, and increased apoptosis in the proximal tubule in early AKI stages (9). Its impact on AKI-to-CKD transition and renal fibrosis remains to be assessed.

Mounting evidence points to the renal microvasculature as a central regulator of fibrosis after IRI (10–12). Increased caspase-3 activation within peritubular capillaries (PTCs) leads to microvascular rarefaction that in turn fuels of persistent renal hypoxia, leading to activation of fibrogenic pathways (11, 13, 14). Caspase-3–deficient mice exposed to renal IRI show reduced PTC rarefaction, decreased fibrosis, and preserved long-term renal function in spite of early increases in TEC necroptosis and early aggravation of renal dysfunction (13, 14). Whether autophagy prevents regulated death of PTCs and impacts renal fibrogenesis has yet to be evaluated.

In this study, we performed detailed assessments of apoptosis, necroptosis, and autophagy over time in tubules and PTCs after renal IRI to tease out their association with renal fibrogenesis. We also evaluated whether interrupting autophagic flux, caspase-3 activation, or both impacts tubular and microvascular regulated cell death and development of renal fibrosis.

## Results

### Renal IRI induces distinct modes of regulated cell death in renal tubular and microvascular compartments.

**Figure 1:**
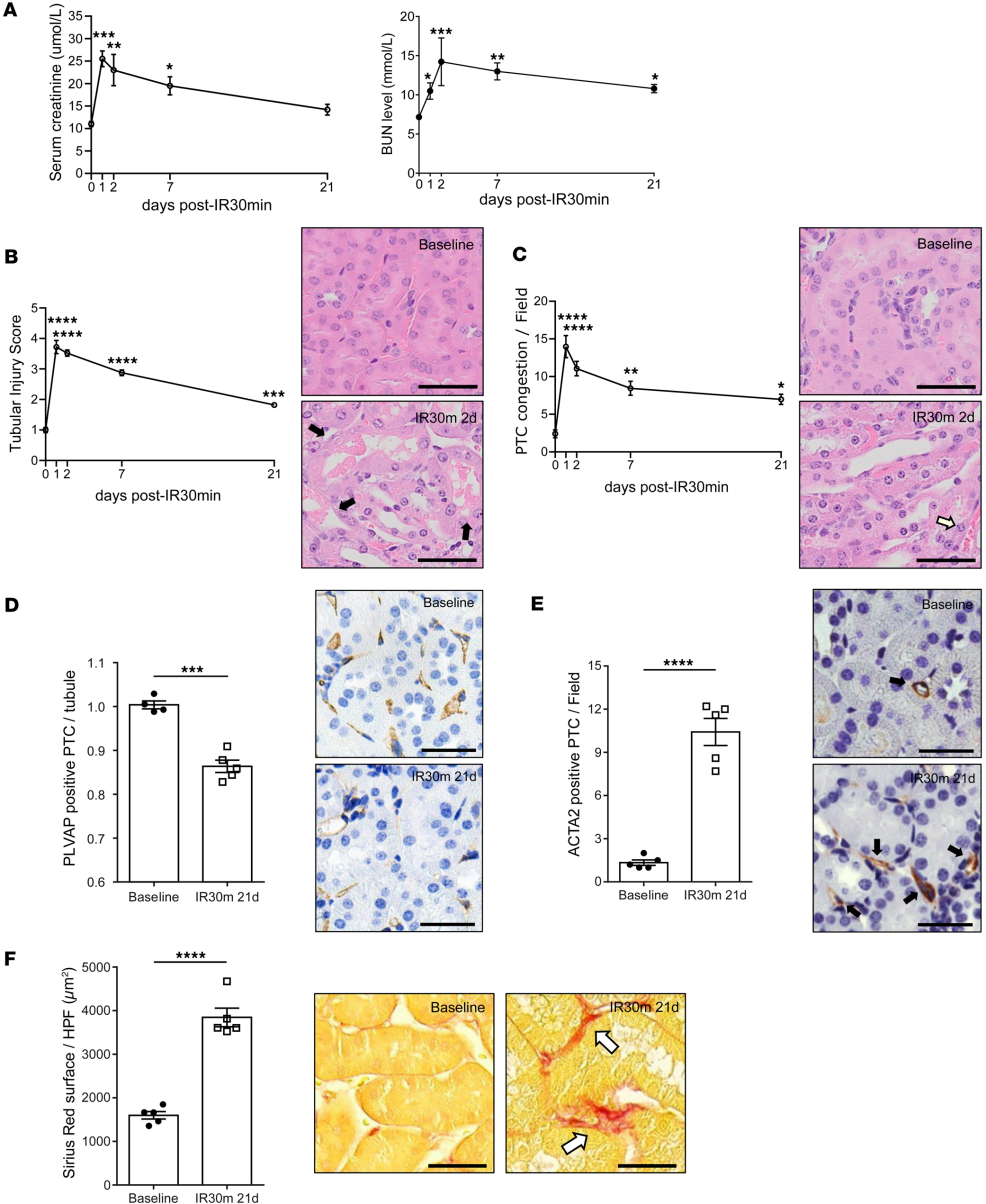
Renal IRI induces kidney dysfunction, tubular damage, microvascular rarefaction, and fibrosis. (**A**) Serum creatinine and BUN levels up to 21 days after renal IRI (*n* = 3–5). IR30min, 30-minute ischemia/reperfusion. (**B**) Mean tubular injury scores in H&E-stained kidney sections (*n* = 4–5). The black arrow indicates injured tubules. (**C**) Mean number of peritubular capillaries (PTCs) with evidence of congestion in H&E-stained kidney sections (*n* = 4–5). The white arrow indicates PTC congestion. (**D**) Quantification of PLVAP immunohistochemistry (IHC) (*n* = 4–5). (**E**) Quantification of ACTA2 IHC (*n* = 5). The black arrow indicates ACTA2^+^ cells. (**F**) Quantification of Sirius red staining (*n* = 5). The white arrow indicates fibrosis. Quantification was performed with samples at baseline, 1, 2, 7, and 21 days after IRI. For each kidney, 10 randomly selected high-power fields (HPFs) (original magnification, ×200) were evaluated, consisting of 5 fields from the cortex and 5 from the corticomedullary junction. All scale bars: 50 μm. Values are mean ± SEM. *P* values obtained by 1-way ANOVA with Bonferroni’s post hoc test (**A**–**C**) or by unpaired, 2-tailed Student’s *t* test (**D**–**F**). *P < 0.05, **P < 0.01, ***P < 0.001, ****P < 0.0001 for comparisons between baseline and each time point.

**Figure 2:**
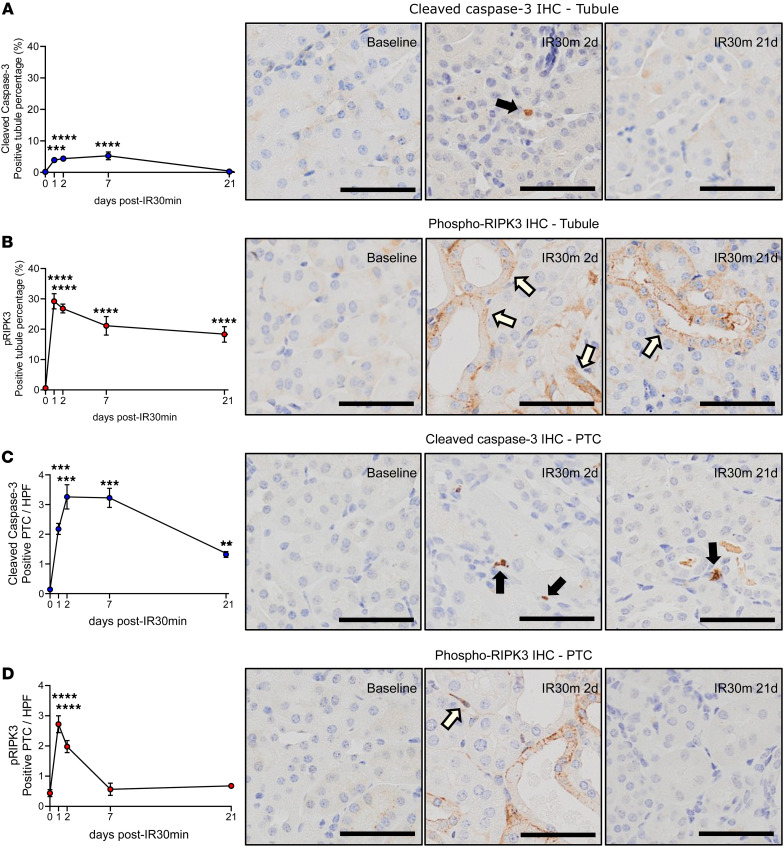
Renal IRI induces unique patterns of caspase-3–dependent apoptosis and necroptosis in peritubular capillaries (PTCs) and tubules. (**A**) Quantification of cleaved caspase-3 immunohistochemistry (IHC) in renal tubules (*n* = 4–5). Representative images of cleaved caspase-3^+^ tubule IHC. IR30min, 30-minute ischemia/reperfusion. (**B**) Quantification of p-RIPK3 IHC in renal tubules (*n* = 3–5). Representative images of p-RIPK3^+^ tubule IHC. (**C**) Quantification of cleaved caspase-3 IHC in renal PTCs (*n* = 4–5). Representative images of cleaved caspase-3^+^ PTC IHC. (**D**) Quantification of p-RIPK3 IHC in renal PTCs (*n* = 3–5). Representative images of p-RIPK3^+^ PTC IHC. All quantifications were performed with samples at baseline and 1, 2, 7, and 21 days after IRI. For each kidney, 10 randomly selected high-power fields (original magnification, ×200) were evaluated, consisting of 5 fields from the cortex and 5 from the corticomedullary junction. The black arrows indicate cleaved caspase-3^+^ cells and the white arrows indicate p-RIPK3^+^ cells. All scale bars: 50 μm. Values are mean ± SEM. *P* values obtained by 1-way ANOVA with Bonferroni’s post hoc test. **P < 0.01, ***P < 0.001, ****P < 0.0001 for comparisons between baseline and each time point.

GFP-LC3 mice were subjected to renal IRI by renal pedicle clamping for 30 minutes and contralateral nephrectomy and followed for up to 21 days. GFP-LC3 mice express a fluorescent form of LC3 for efficient monitoring of LC3^+^ autophagosomes. Serum creatinine levels increased significantly during the acute phase after IRI and gradually decreased until day 21 (Figure 1A and Supplemental Figure 1, A and B; supplemental material available online with this article; https://doi.org/10.1172/jci.insight.201945DS1). Blood urea nitrogen (BUN) levels showed a similar trend (Figure 1A). Significant tubular damage and PTC congestion were present at day 1 and persisted until day 21 after IRI (Figure 1, B and C, and Supplemental Figure 1, C and D). At 21 days after IRI, significantly reduced plasmalemma vesicle–associated protein (PLVAP) staining within the peritubular space confirmed PTC rarefaction. Accumulation of interstitial α-smooth muscle actin^+^ (ACTA2^+^) cells and increased collagen deposition confirmed development of renal fibrosis (Figure 1, D–F). These findings highlight the presence of persistent renal injury despite amelioration of serum creatinine and BUN levels, consistent with a large body of evidence supporting the notion that creatinine and BUN are not sensitive biomarkers of renal fibrosis (15, 16). We then assessed the relative contributions of necroptosis and apoptosis to tubular and PTC cell death over time. Tubules exhibited a transient and significant increase in activated caspase-3 at 1, 2, and 7 days after IRI, returning to baseline levels by day 21 (Figure 2A and Supplemental Figure 2A). In contrast, phospho-RIPK3 (p-RIPK3), a marker of necroptosis, was increased in renal tubules at 1 and 2 days after IRI and remained elevated through day 21 (Figure 2B and Supplemental Figure 2B). In PTCs, both apoptosis and necroptosis were activated during the acute phase, with significant increases in cleaved caspase-3 and p-RIPK3 observed at 1 and 2 days after IRI. However, while p-RIPK3 levels returned to baseline by day 7, cleaved caspase-3 continued to show significant increases through day 21 (Figure 2, C and D, and Supplemental Figure 2, C and D).

**Figure 3:**
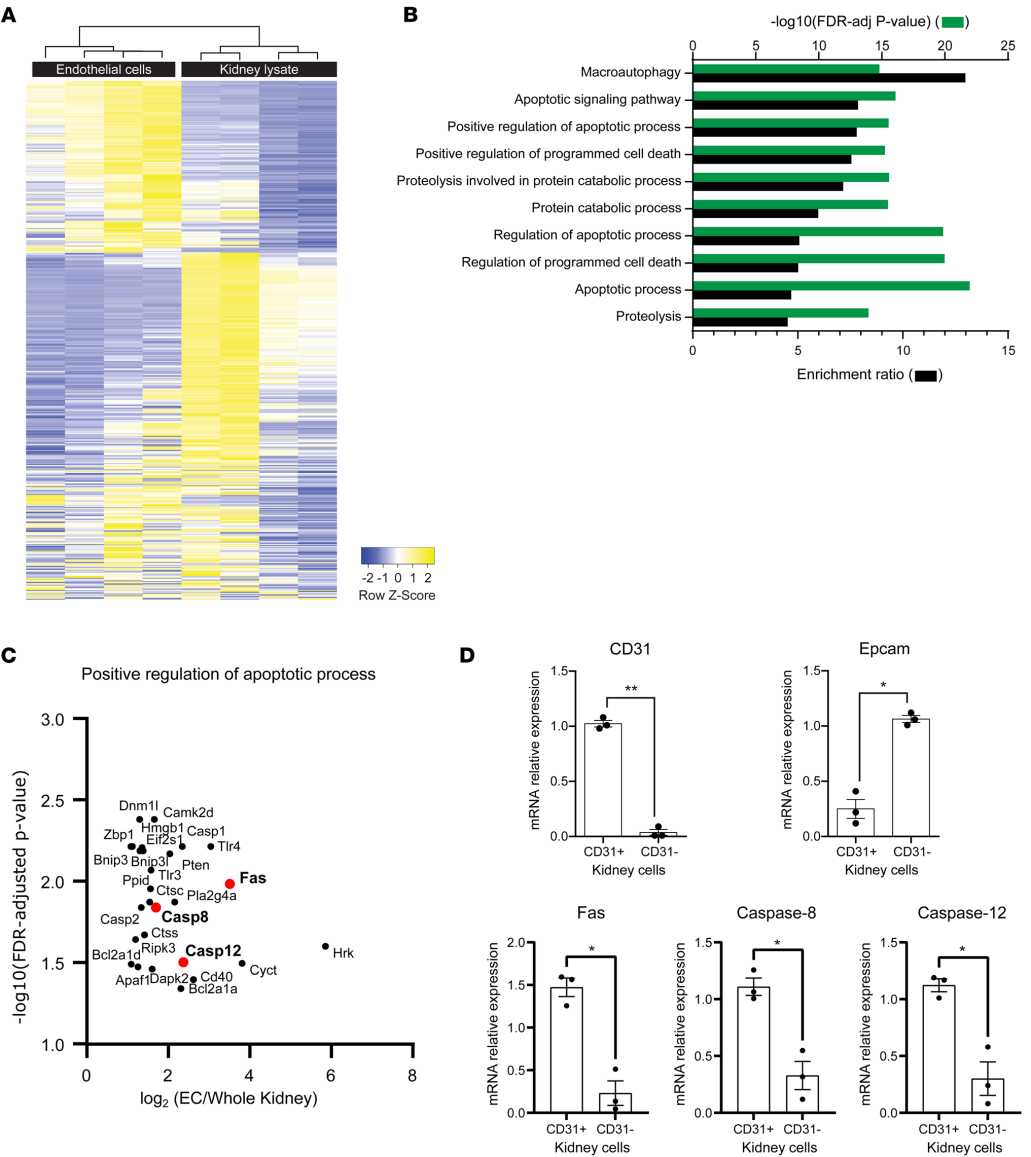
Renal endothelial cells exhibit a unique mRNA expression profile related to cell death compared with whole kidney lysates. (**A**) Heatmap illustrating the expression of genes involved in apoptosis, necroptosis, and autophagy (derived from the KEGG and Reactome pathways) in renal endothelial cells and kidney lysate groups; *n* = 4. (**B**) Biological process enrichment analysis of transcripts differentially expressed in renal endothelial cells compared with the whole kidney lysate. An FDR-adjusted *P* value of less than 0.05 was considered significant. (**C**) Volcano plot of transcripts identified in the biological process “Positive regulation of apoptotic process” and enriched in kidney endothelial cells (EC). The plot displays the significantly differentially expressed transcripts identified through transcriptomic analysis. Transcripts are ranked in the volcano plot according to their FDR-adjusted *P* value (*y* axis) as –log_10_ and their relative enrichment ratio (log_2_) between endothelial cells and whole kidney lysate (*x* axis). Red dots represent the transcripts selected for validation by quantitative RT-PCR. (**D**) CD31^+^ and CD31^–^ cells were isolated from whole kidney. The expression of *CD31*, *Epcam*, *Fas*, *Caspase-8*, and *Caspase-12* mRNAs was measured by quantitative RT-PCR. The results are presented as the relative expression of mRNA ± SEM after normalization to *Hprt1*; *n* = 3 for each group. *P* values were obtained by unpaired, 2-tailed Student’s *t* test. *P < 0.05, **P < 0.01.

This suggested that PTCs and TECs have a distinct propensity for regulated cell death pathways. To test this possibility, we compared basal expression of apoptosis- and necroptosis-regulating genes in renal endothelial and non-endothelial renal cells using publicly available transcriptomic data from the NCBI Gene Expression Omnibus (GEO) database (accession number GSE300619) (17). Hierarchical clustering and Gene Ontology (GO) analyses revealed that renal endothelial cells have a unique mRNA expression profile related to cell death compared with whole kidney lysates (mainly composed of TECs). This profile shows significant enrichment of biological processes related to the positive regulation of apoptosis and macroautophagy (Figure 3, A–C). To confirm these findings in our animal model, we freshly isolated renal CD31^+^ and CD31^–^ cells from GFP-LC3 mice at baseline. To confirm cell enrichment in our CD31^+^ and CD31^–^ fractions, we assessed the expression of the endothelial marker CD31 and the epithelial marker Epcam by quantitative PCR. As expected, the CD31^+^ fraction showed marked enrichment of CD31, whereas the CD31^–^ fraction was enriched in Epcam (Figure 3D). Following this validation, we evaluated the expression of 3 apoptosis-regulating genes (*Caspase-8*, *Caspase-12*, and *Fas*) identified as differentially overexpressed (log_2_[fold change] > 1.5 and –log_10_[FDR-adjusted *P* value] > 1.5) in the publicly available transcriptomic data (Figure 3C). PCR analysis confirmed significantly higher basal expression of all 3 genes in the renal endothelial fraction (Figure 3D). These results show that renal endothelial cells have a basal expression pattern of apoptosis-regulating genes that differs fundamentally from that of TECs and likely supports distinct propensity for regulated death pathways. Collectively, these results support the notion that IRI induces prolonged renal damage through distinct modes of regulated cell death, with renal tubules predominantly engaging in necroptosis and PTCs in caspase-3–dependent apoptosis.

### Autophagy dynamics in tubules and PTCs after renal IRI.

**Figure 4:**
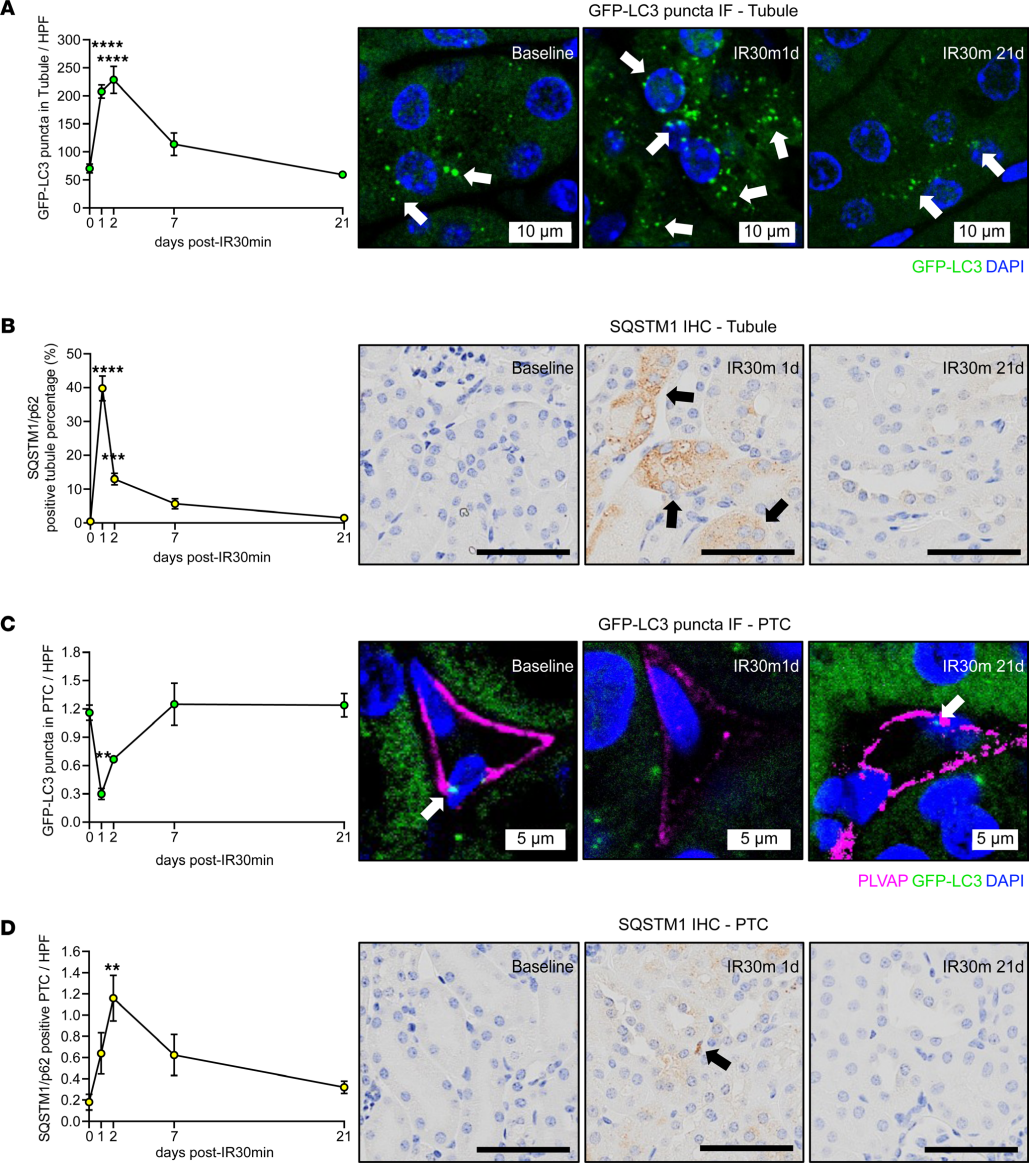
Renal IRI induces autophagy in tubules and peritubular capillaries (PTCs). (**A**) Quantification of GFP-LC3^+^ puncta in renal tubules (*n* = 4–5). Representative images of autophagic puncta in tubules. IR30min, 30-minute ischemia/reperfusion. (**B**) Quantification of SQSTM1/p62 immunohistochemistry (IHC) in renal tubules (*n* = 4–5). Representative images of SQSTM1/p62 IHC in tubules. (**C**) Quantification of autophagic puncta in renal PTCs (*n* = 3–5). Representative images of autophagic puncta in PTCs. (**D**) Quantification of SQSTM1/p62 IHC in renal PTCs (*n* = 4–5). Representative images of SQSTM1/p62 IHC in PTCs. All quantifications were performed with samples at baseline, 1, 2, 7, and 21 days after IRI. For each kidney, 10 randomly selected high-power fields (original magnification, ×200) were evaluated, consisting of 5 fields from the cortex and 5 from the corticomedullary junction. Immunofluorescence (IF) staining for GFP-LC3 (green), PLVAP (magenta), and DAPI (blue). The white arrows indicate GFP-LC3^+^ puncta and the black arrows indicate SQSTM1/p62^+^ cells. Scale bars: 50 μm (IHC), 10 μm (IF, tubule), and 5 μm (IF, PTC). Values are mean ± SEM. *P* values obtained by 1-way ANOVA with Bonferroni’s post hoc test. **P < 0.01, ***P < 0.001, ****P < 0.0001 for comparisons between baseline and each time point.

We then investigated the time course of autophagy activation in renal tubules and PTCs following IRI. Induction of autophagy is classically associated with increased numbers of GFP-LC3^+^ puncta and degradation of the sequestosome 1 (SQSTM1/p62) (18). Renal tubules showed an increased number of GFP-LC3^+^ puncta at 1 and 2 days after IRI, followed by a decrease until day 21 (Figure 4A and Supplemental Figure 3A). SQSTM1/p62 levels in tubules were significantly increased on day 1 after IRI, decreased at day 2, and continued to decrease until day 21 (Figure 4B and Supplemental Figure 3B). These results suggest that autophagy increases in tubules during the first 2 days after IRI and reverts to baseline by day 21 (Figure 4, A and B). Western blot analysis of whole kidney lysates for LC3 and SQSTM1/p62 revealed similar trends to those observed in tubules (Supplemental Figure 4). In PTCs, the number of GFP-LC3^+^ puncta was significantly decreased on day 1 after IRI, followed by an increase up to day 7 and maintenance at this level until day 21 (Figure 4C and Supplemental Figure 3C). SQSTM1/p62 levels exhibited an early increase followed by a progressive decline until day 21 (Figure 4D and Supplemental Figure 3D). These results suggest that, in PTCs, autophagy is inhibited in early stages of AKI, followed by progressive resumption of autophagic activity.

**Figure 5:**
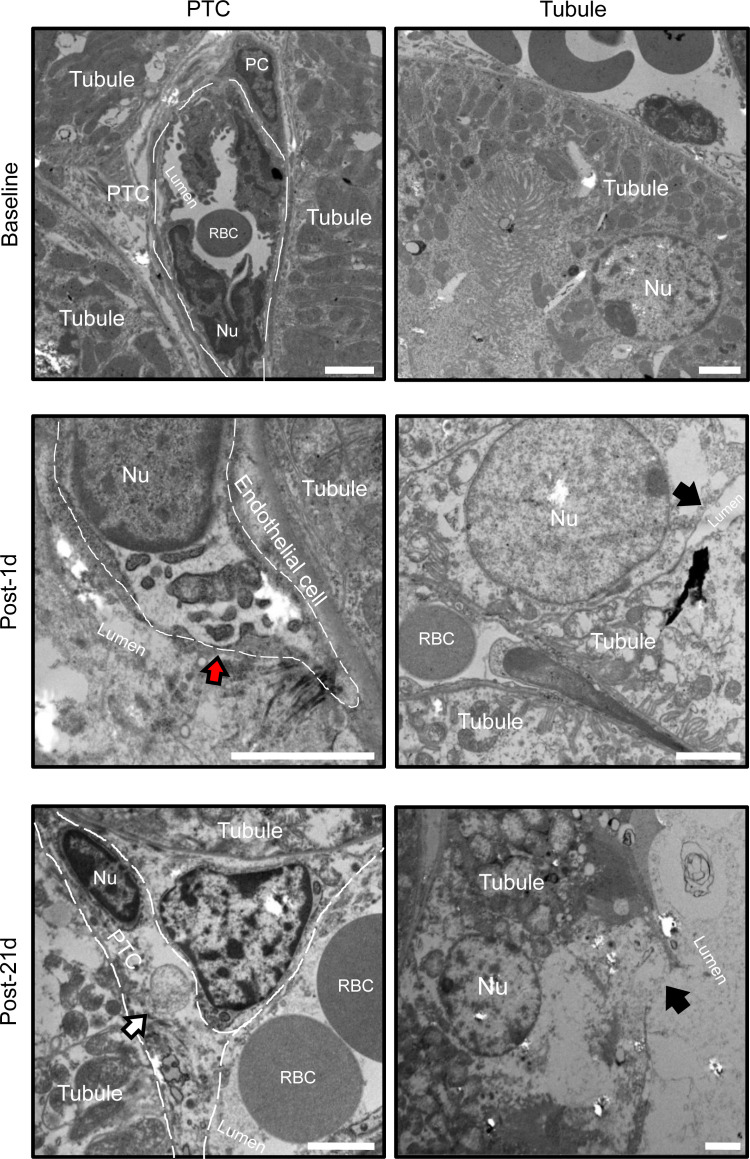
Caspase-3–dependent apoptosis, necroptosis, and autophagy in renal tubules and PTCs following renal IRI. Representative transmission electron microscopy image shows normal PTC and tubular ultrastructure at baseline as well as predominant programmed cell death (PCD) and autophagy in renal tubules and PTC at 1 and 21 days after IRI. The black arrows indicate cytosol rupture towards the tubular lumen, the red arrow apoptotic bodies, and the white arrow autolysosomes. Scale bars: 2 μm.

To confirm these findings, we evaluated the ultrastructure of tubules and PTCs after renal IRI by electron microscopy (Figure 5). At day 1 after IRI, damaged tubules exhibited necrotic features with nuclear enlargement, damaged mitochondria, and cytosolic rupture extending into the tubular lumen. These changes persisted until day 21. Damaged PTCs showed morphological features of apoptosis activation after IRI, as evidenced by the secretion of apoptotic bodies. Evidence of autophagic activation with the formation of large autolysosomes was also found at 21 days after IRI in PTCs. Taken together, these results confirm that after renal IRI, tubules primarily undergo necroptosis, which is sustained over time. In contrast, apoptosis is only transiently activated early on, and autophagy peaks early and briefly before declining. Conversely, PTCs exhibit early necroptosis with rapid resumption, whereas apoptosis is rapidly activated and increases over time and autophagy gradually intensifies after an early blockade after renal IRI.

### Autophagic flux disruption aggravates renal microvascular injury and fibrosis.

**Figure 6:**
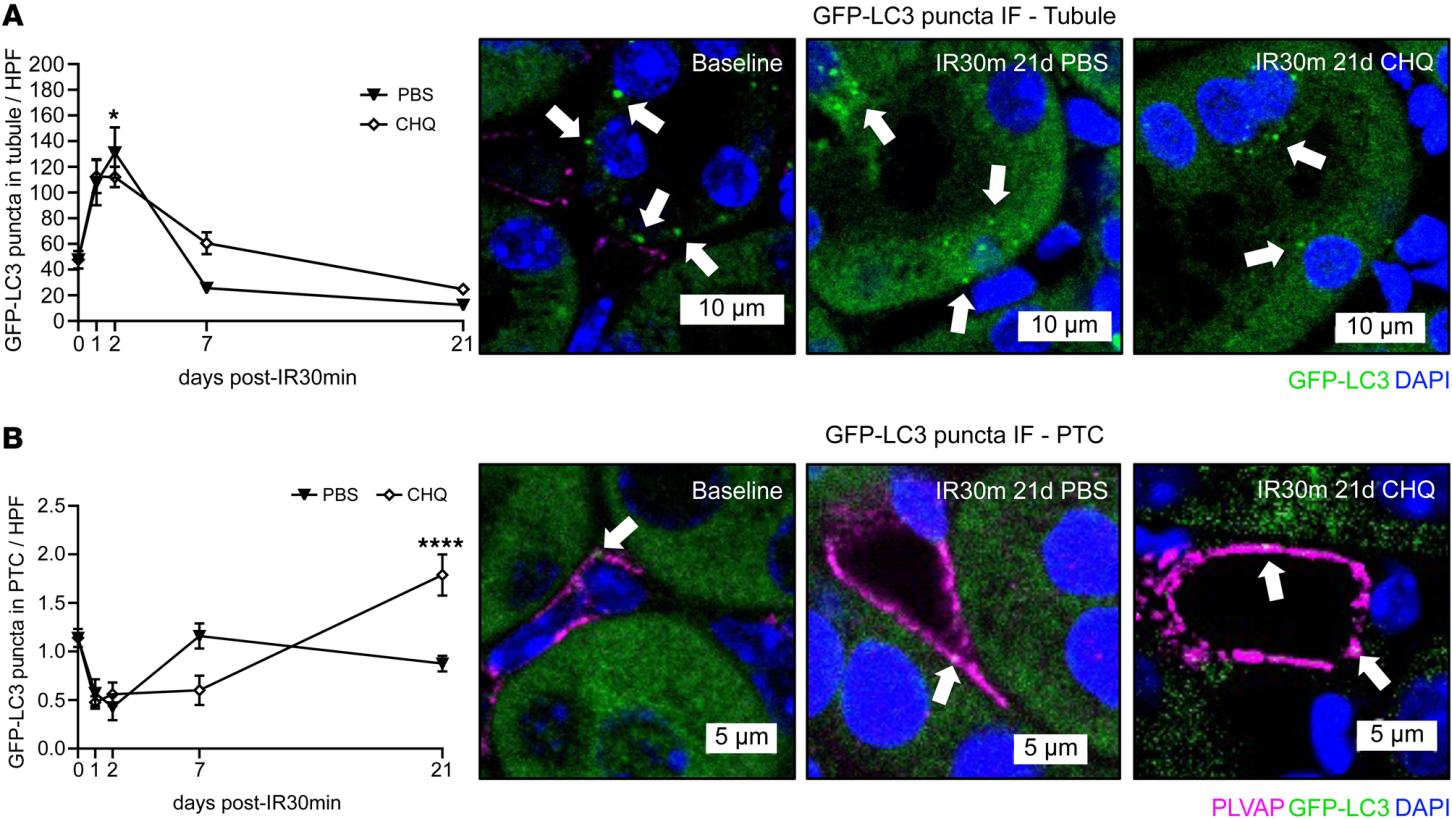
Chloroquine (CHQ) inhibits autophagy following renal IRI. (**A**) Quantification of GFP-LC3^+^ puncta in renal tubules from PBS- and CHQ-injected mice (*n* = 4–6). Representative images of autophagic puncta in tubules. IR30min, 30-minute ischemia/reperfusion. (**B**) Quantification of autophagic puncta in renal peritubular capillaries (PTCs) from PBS- and CHQ-injected mice (*n* = 4–8). Representative images of autophagic puncta in PTCs. All quantifications were performed with samples at baseline and 1, 2, 7, and 21 days after IRI. For each kidney, 10 randomly selected high-power fields (original magnification, ×200) were evaluated, consisting of 5 fields from the cortex and 5 from the corticomedullary junction. Immunofluorescence (IF) staining for GFP-LC3 (green), PLVAP (magenta), and DAPI (blue). The white arrow indicates GFP-LC3^+^ puncta. Scale bars: 10 μm (IF, tubule) and 5 μm (IF, PTC). Values are mean ± SEM. *P* values obtained by 1-way ANOVA with Bonferroni’s post hoc test. *P < 0.05, ****P < 0.0001 for comparisons between PBS and CHQ at the same time points.

**Figure 7:**
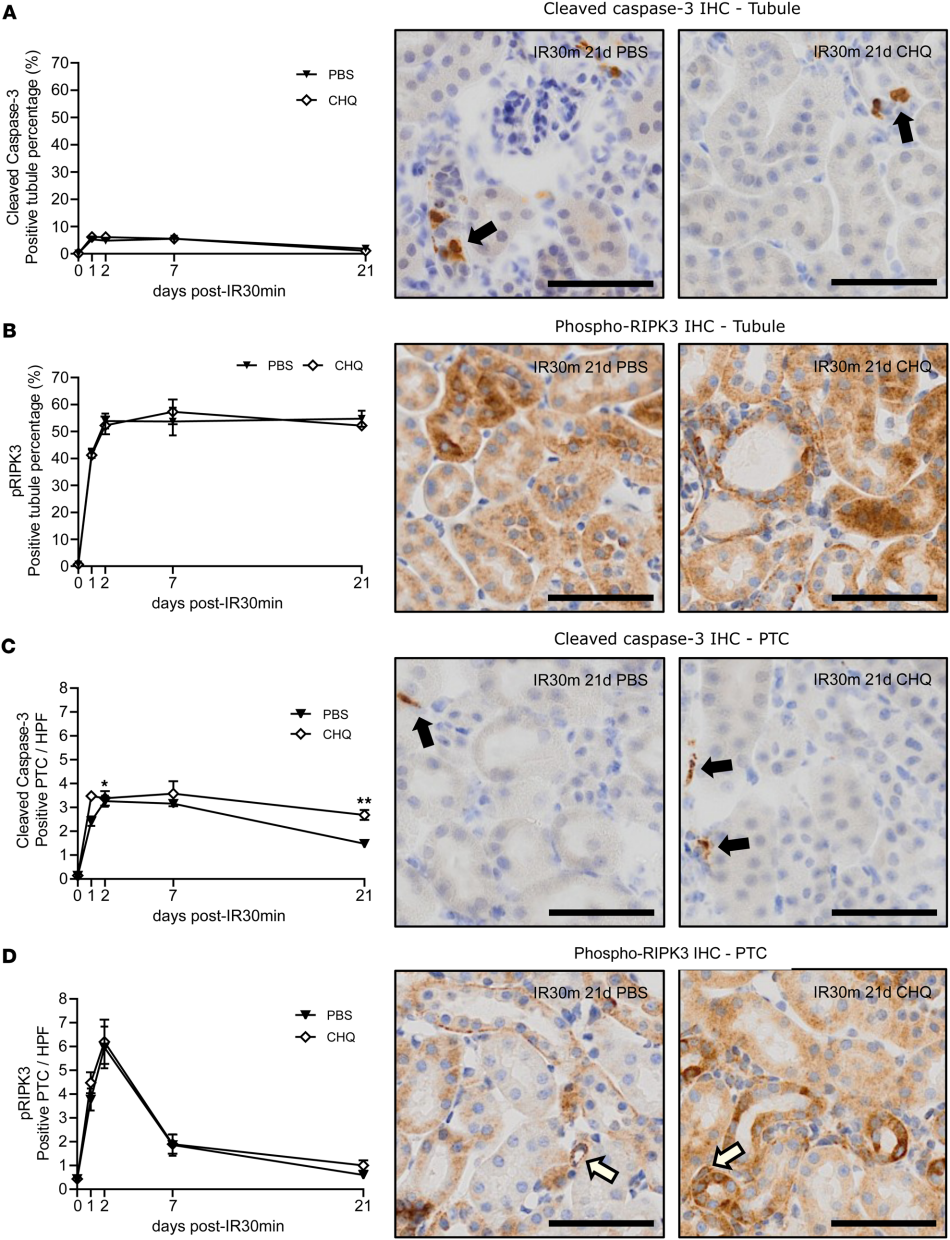
Autophagy disruption enhances apoptosis of peritubular capillaries (PTCs) following renal IRI. (**A**) Quantification of cleaved caspase-3 immunohistochemistry (IHC) in renal tubules from PBS- and chloroquine-injected (CHQ-injected) mice (*n* = 4–6). Representative images of cleaved caspase-3 IHC in tubules. IR30min, 30-minute ischemia/reperfusion. (**B**) Quantification of p-RIPK3 IHC in renal tubules from PBS- and CHQ-injected mice (*n* = 3–6). Representative images of p-RIPK3 IHC in tubules. (**C**) Quantification of cleaved caspase-3 IHC in renal PTCs from PBS- and CHQ-injected mice (*n* = 4–6). Representative images of cleaved caspase-3 IHC in PTCs. (**D**) Quantification of p-RIPK3 IHC in renal PTCs from PBS- and CHQ-injected mice (*n* = 4–6). Representative images of p-RIPK3 IHC in PTCs. All quantifications were performed with samples at baseline and 1, 2, 7, and 21 days after IRI. For each kidney, 10 randomly selected high-power fields (HPFs) (original magnification, ×200) were evaluated, consisting of 5 fields from the cortex and 5 from the corticomedullary junction. The black arrows indicate cleaved caspase-3^+^ cells and the white arrows indicate p-RIPK3^+^ cells. All scale bars: 50 μm. Values are mean ± SEM. *P* values obtained by 1-way ANOVA with Bonferroni’s post hoc test. *P < 0.05, **P < 0.01 for comparisons between PBS and CHQ at the same time points.

We then used chloroquine (CHQ) administration to evaluate the functional impact of autophagy inhibition on the tubular and PTC compartments after renal IRI. We first assessed kidney dysfunction and observed that autophagy inhibition with CHQ significantly increased serum creatinine levels on day 2 after IRI (Supplemental Figure 5). CHQ inhibits autophagic flux by preventing the fusion of autophagosomes with lysosomes, which translates into intracellular accumulation of LC3^+^ puncta that cannot be degraded by lysosomes (19). CHQ-injected mice showed increased numbers of GFP-LC3^+^ puncta at day 7 in renal tubules (Figure 6A and Supplemental Figure 6A) and, in PTCs, increased numbers of GFP-LC3^+^ puncta at day 21 (Figure 6B and Supplemental Figure 6B); again, suggesting that resumption of autophagy after IRI occurs at later time points in PTCs compared with tubules. Next, we evaluated the effects of CHQ on apoptosis and necroptosis over time in renal tubules and PTCs. CHQ did not modulate levels of cleaved caspase-3 or p-RIPK3 in tubules after IRI (Figure 7, A and B, and Supplemental Figure 7, A and B). In contrast, autophagy disruption led to a significant increase in cleaved caspase-3 levels in PTCs at both 1 and 21 days after IRI (Figure 7C and Supplemental Figure 7C), while p-RIPK3 levels remained unaffected (Figure 7D and Supplemental Figure 7D). These results highlight evidence of crosstalk between autophagic and apoptotic pathways in PTCs but not in TECs.

**Figure 8:**
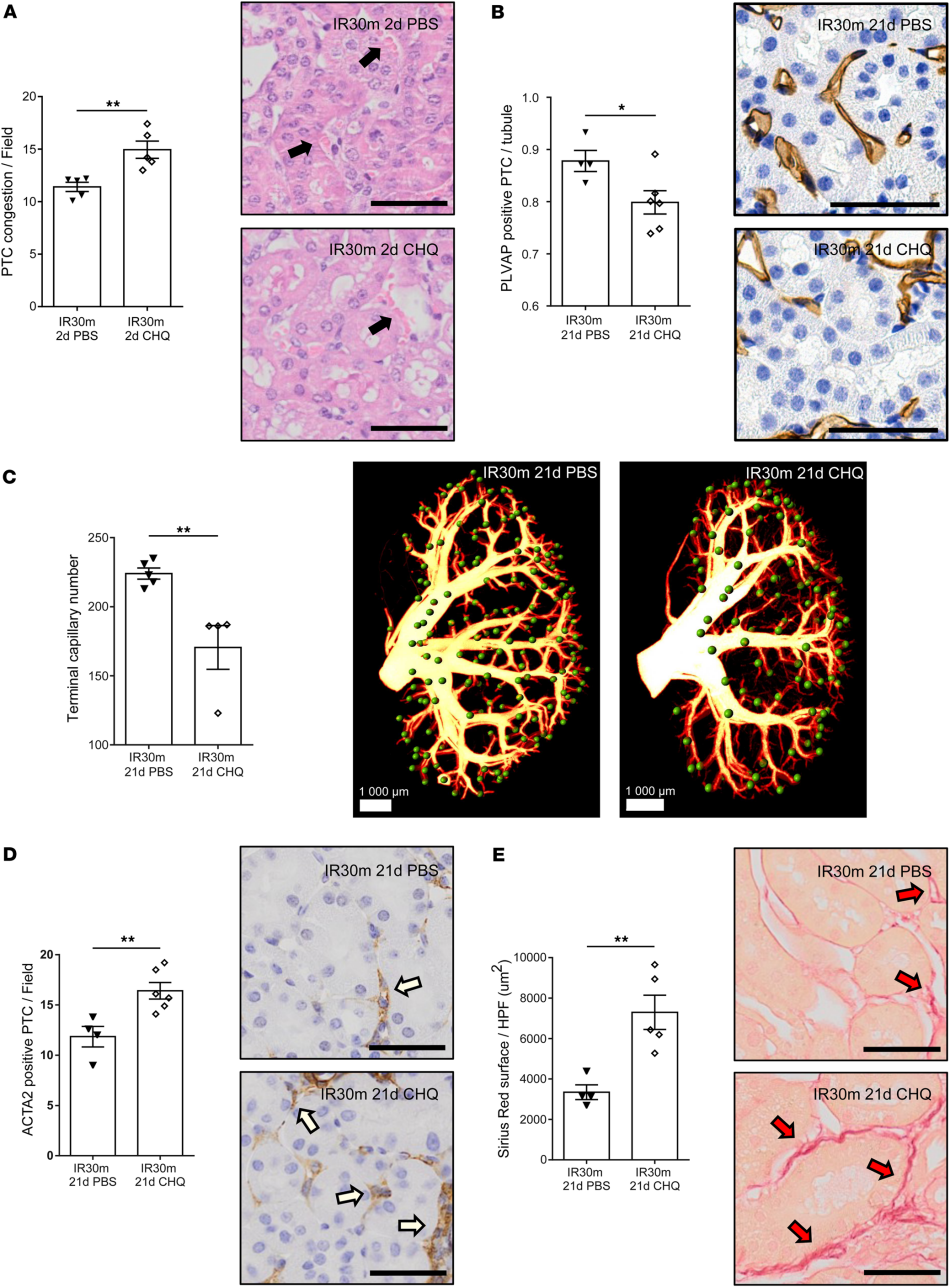
Autophagy disruption aggravates microvascular rarefaction and fibrosis following renal IRI. (**A**) Quantification of peritubular capillary (PTC) congestion in H&E-stained kidney sections from GFP-LC3 mice injected with PBS or chloroquine (CHQ) at 2 days after IRI (*n* = 5). Representative images of H&E staining. The black arrows indicate PTC congestion. IR30m, 30-minute ischemia/reperfusion. (**B**) Quantification of PLVAP immunohistochemistry (IHC) in the renal tissues from GFP-LC3 mice injected with PBS or CHQ at 21 days after IRI (*n* = 4–6). Representative images of PLVAP IHC. (**C**) Quantification of terminal capillary numbers in the whole kidney (*n* = 4–5). 3D reconstruction images of the entire renal microvasculature using representative microCT from PBS- or CHQ-treated mice at 21 days after IRI. (**D**) Quantification of α-smooth muscle actin (ACTA2) IHC in the kidney from PBS- and CHQ-injected mice at 21 days after IRI (*n* = 4–6). Representative images of ACTA2 IHC. The white arrows indicate ACTA2^+^ cells. (**E**) Quantification of Sirius red staining in the kidney from PBS- and CHQ-injected mice at 21 days after IRI (*n* = 4–5). Representative images of Sirius red staining. The red arrows indicate collagen deposition. For each kidney, 10 randomly selected high-power fields (original magnification, ×200) were evaluated, consisting of 5 fields from the cortex and 5 from the corticomedullary junction. Scale bars: 50 μm (H&E, IHC, and Sirius red) and 1000 μm (microCT 3D recon image). Values are mean ± SEM. *P* values were obtained by unpaired, 2-tailed Student’s *t* test. *P < 0.05, **P < 0.01 for comparisons between PBS- and CHQ-treated mice at 2 days (**A**) or 21 days (**B**–**E**) after IRI.

Caspase-3–dependent PTC death leading to microvascular rarefaction and establishment of a hypoxic microenvironment is a critical determinant of renal fibrosis after IRI (14, 20, 21). Therefore, we investigated whether increased caspase-3 activation in PTCs with CHQ treatment affects microvascular integrity and fibrosis after IRI. CHQ exacerbated PTC congestion 2 days after IRI (Figure 8A). At 21 days, CHQ treatment led to aggravated PTC rarefaction with decreased PLVAP staining (Figure 8B). Contrast-enhanced microcomputed tomography (microCT) confirmed enhanced microvascular rarefaction, with a lower number of terminal capillaries in CHQ-treated mice compared with the control group (Figure 8C). Markers of renal fibrosis were also increased in CHQ-treated mice on day 21 after IRI, with increased interstitial ACTA2 staining (Figure 8D) and Sirius red staining (Figure 8E). Taken together, these results show that disruption of the autophagic flux does not significantly impact tubular injury but exacerbates PTC apoptosis and PTC rarefaction as well as renal fibrogenesis.

### Crosstalk between autophagic and caspase-3–dependent pathways in PTCs controls renal fibrosis.

**Figure 9:**
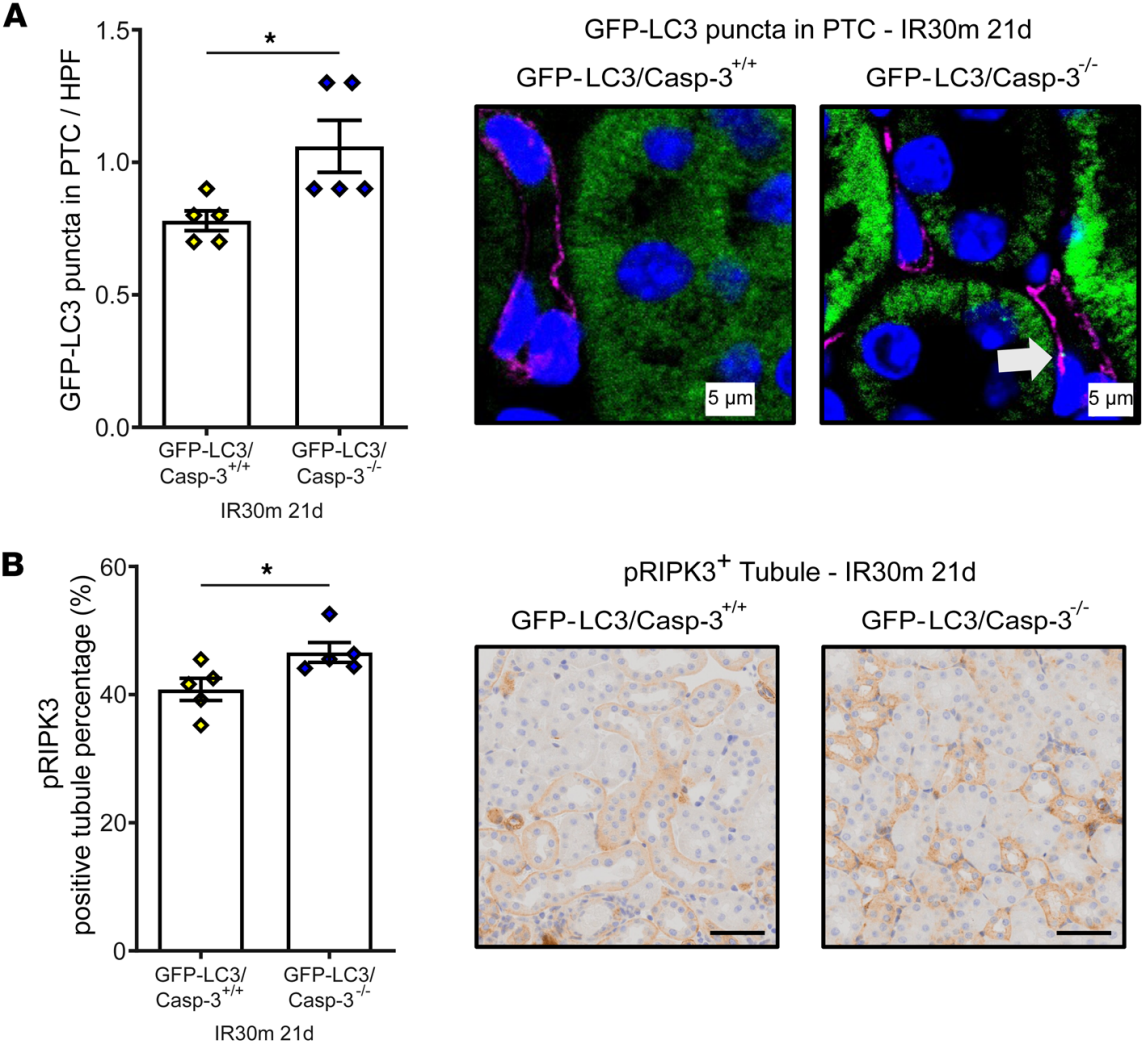
Concomitant autophagy disruption and caspase-3 deficiency enhance necroptosis in peritubular capillaries (PTCs) following renal IRI. (**A**) Quantification of GFP-LC3^+^ puncta in PTCs of GFP-LC3/*Caspase-3^+/+^* and GFP-LC3/*Caspase-3^–/–^* mouse kidneys (*n* = 5). Representative images of autophagic puncta in renal PTCs. Immunofluorescence (IF) staining for GFP-LC3 (green), PLVAP (magenta), and DAPI (blue). The white arrows indicate GFP-LC3^+^ puncta. IR30m, 30-minute ischemia/reperfusion. (**B**) Quantification of p-RIPK3 IHC in renal PTCs of GFP-LC3/*Caspase-3^+/+^* and GFP-LC3/*Caspase-3^–/–^* mouse kidneys (*n* = 5). Representative images of p-RIPK3 IHC in renal PTCs. For each kidney, 10 randomly selected high-power fields (original magnification, ×200) were evaluated, consisting of 5 fields from the cortex and 5 from the corticomedullary junction. Scale bars: 50 μm (IHC) and 5 μm (IF). Values are mean ± SEM. *P* values were obtained by unpaired, 2-tailed Student’s *t* test. *P < 0.05 for comparisons between PBS- and CHQ-treated mice at 21 days after IRI.

We previously demonstrated, using *Caspase-3^–/–^* mice, that caspase-3 controls renal microvascular rarefaction and fibrosis after IRI (13). Building on these findings, we explored the interaction between autophagy and caspase-3–dependent apoptosis after renal IRI using GFP-LC3/*Caspase-3^–/–^* mice and GFP-LC3/*Caspase-3^+/+^* control littermates. GFP-LC3/*Caspase-3^–/–^* mice showed higher levels of GFP-LC3^+^ puncta at 21 days after IRI compared with GFP-LC3/*Caspase-3^+/+^* (Figure 9A). This result is consistent with previous work showing a role for caspase-3 in fusion of autolysosomes with the endothelial cell membrane (22). GFP-LC3/*Caspase-3^–/–^* mice showed increased tubular p-RIPK3 staining when compared with GFP-LC3/*Caspase-3^+/+^* mice (Figure 9B), confirming enhanced tubular necroptosis in the presence of caspase-3 invalidation. Yet, enhanced tubular necroptosis was not associated with enhanced fibrosis, as GFP-LC3/*Caspase-3^–/–^* mice showed reduced renal fibrosis with lower levels of ACTA2^+^ cells and lower collagen deposition in the interstitial space (Supplemental Figure 8A-B). GFP-LC3/*Caspase-3^–/–^* mice showed increased microvascular PTC density at 21 days after IRI when compared with GFP-LC3/*Caspase-3^+/+^* (Supplemental Figure 8C), confirming the association between preservation of microvascular density and prevention of fibrosis.

**Figure 10:**
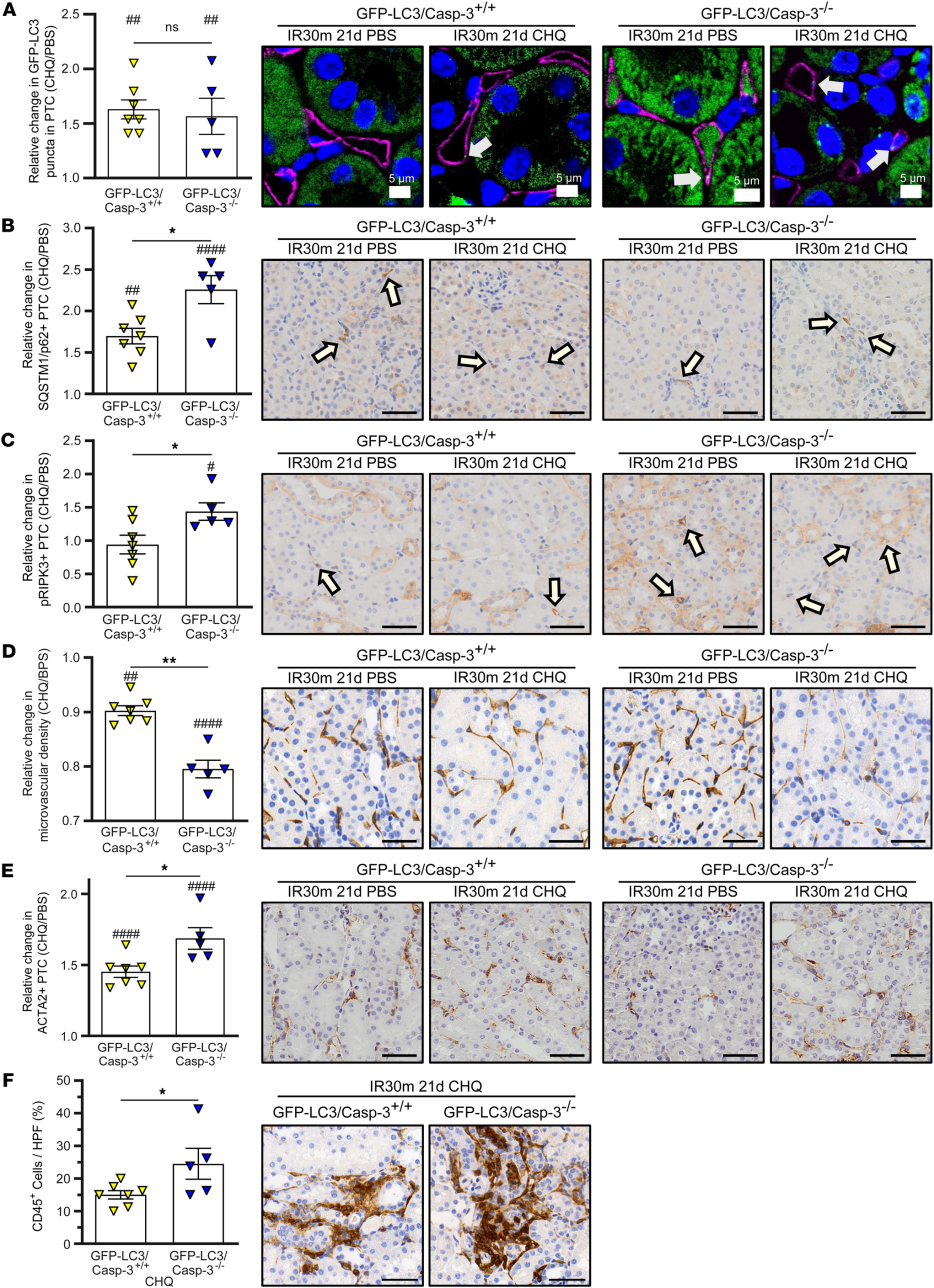
Autophagy disruption and caspase-3 deficiency worsen microvascular rarefaction, fibrosis, and leukocyte infiltration following renal IRI. (**A**–**E**) Quantification of the relative change in specific markers within peritubular capillaries (PTCs) of GFP-LC3/*Caspase-3^+/+^* and GFP-LC3/*Caspase-3^–/–^* mice injected with chloroquine (CHQ) compared to those injected with PBS (*n* = 5–7 per group). (**A**) Relative change in GFP-LC3^+^ puncta. Representative images show autophagic puncta (white arrows) in renal PTCs using immunofluorescence (IF) staining for GFP-LC3 (green), PLVAP (magenta), and DAPI (blue). IR30m, 30-minute ischemia/reperfusion. (**B**) Relative change in SQSTM1/p62^+^ PTCs. Representative images show SQSTM1/p62 immunohistochemistry (IHC) in PTCs, with white arrows indicating SQSTM1/p62^+^ cells. (**C**) Relative change in p-RIPK3 IHC in PTCs. Representative images show p-RIPK3 IHC in PTCs, with white arrows indicating p-RIPK3^+^ PTCs. (**D**) Relative change in microvascular density. Representative images show PLVAP IHC in kidney tissue. (**E**) Relative change in α-smooth muscle actin^+^ (ACTA2^+^) PTCs. Representative images show ACTA2 IHC in PTCs, with white arrows indicating ACTA2^+^ cells. (**F**) Quantification of the ratio of CD45^+^ area to total tissue area (*n* = 5–7). Representative images of the CD45^+^ area. For each kidney, 10 randomly selected high-power fields (original magnification, ×200) were evaluated, consisting of 5 fields from the cortex and 5 from the corticomedullary junction. Scale bars: 50 μm (IHC) and 5 μm (IF). Values are mean ± SEM. *P* values obtained by 1-way ANOVA with Bonferroni’s post hoc test. ^#^*P* < 0.05, ^##^*P* < 0.01, ^####^*P* < 0.0001 compared with the respective PBS-treated group; *P < 0.05, **P < 0.01 compared with the CHQ-injected group.

We then evaluated whether disruption of autophagy with CHQ would modulate kidney dysfunction, microvascular rarefaction, and development of fibrosis in caspase-3–deficient mice. CHQ-treated GFP-LC3/*Caspase-3^+/+^* littermates exhibited a significant increase in serum creatinine levels at 2 days after surgery; however, this effect was not observed in GFP-LC3/*Caspase-3^–/–^* mice (Supplemental Figure 9A). Next, we assessed kidney injury molecule-1 (KIM-1) expression as a marker of tubular injury. Although KIM-1 levels increased in the wild-type littermate group at 2 days after IRI compared with baseline levels, no significant differences were observed between the PBS- and CHQ-treated groups, or between the two transgenic lines (Supplemental Figure 9, B and C). CHQ injection enhanced the accumulation of GFP-LC3^+^ puncta in PTCs both in GFP-LC3/*Caspase-3^+/+^* and GFP-LC3/*Caspase-3^–/–^* mice. CHQ injection was also associated with increased numbers of SQSTM1/p62^+^ PTCs, together confirming blockade of the autophagic flux (Figure 10, A and B, Supplemental Figure 10, A and B). CHQ administration enhanced PTC caspase-3 activation in GFP-LC3/*Caspase-3^+/+^* mice at 21 days after IRI compared with PBS-treated mice, whereas GFP-LC3/*Caspase-3^–/–^* mice did not express caspase-3 in PTCs (Supplemental Figure 10C). Levels of p-RIPK3 in PTCs were increased in GFP-LC3/*Caspase-3^–/–^* mice when compared with GFP-LC3/*Caspase-3^+/+^* mice at 2 and 21 days after IRI, only in mice administered with CHQ (Figure 10C and Supplemental Figure 10, D–F). These findings suggest that, in PTCs, concomitant autophagy and apoptosis inhibition are required to activate necroptosis.

We then evaluated the impact of concurrent caspase-3 deficiency and autophagy disruption on microvascular rarefaction and renal fibrosis. The loss of PTCs at day 21 after IRI was greater in CHQ-treated GFP-LC3/*Caspase-3^–/–^* mice compared with CHQ-treated GFP-LC3/*Caspase-3^+/+^* mice (Figure 10D and Supplemental Figure 10G), suggesting that activation of necroptosis led to greater microvascular rarefaction. The increase in ACTA2^+^ cells observed in GFP-LC3/*Caspase-3^–/–^* mice treated with CHQ was also greater than that of CHQ-treated GFP-LC3/*Caspase-3^+/+^* mice (Figure 10E and Supplemental Figure 10H). A similar trend was observed for Sirius red staining but did not reach statistical significance (Supplemental Figure 10, I and J). As necroptosis is known as a proinflammatory type of cell death, we then evaluated whether microvascular necroptosis was associated with increased leukocyte infiltration. GFP-LC3/*Caspase-3^–/–^* mice treated with CHQ showed increased leukocyte infiltration when compared with CHQ-treated GFP-LC3/*Caspase-3^+/+^* mice (Figure 10F and Supplemental Figure 10K). These results suggest that increased PTC necroptosis in CHQ-treated GFP-LC3/*Caspase-3^–/–^* mice is associated with increased and persistent renal inflammation as well as increased microvascular rarefaction, both contributing to enhanced renal fibrosis.

## Discussion

Multiple cell death and stress pathways are activated in kidneys following IRI (7, 23–25) and can contribute to maladaptive repair pathways leading to renal fibrosis. The present work enhances our understanding of the cell-type-specific contributions of these various mechanisms. Using an in vivo model of IRI with different transgenic mouse models and pharmacological approaches, we demonstrate that renal IRI induces distinct patterns of autophagy and programmed cell death (PCD) over time in TECs and PTCs. While necroptosis predominates in TECs throughout all stages of AKI-to-CKD transition, caspase-3–dependent apoptosis emerges as the preferred and sustained death pathway in PTCs. Unlike TECs, PTCs exhibit limited regenerative capacity, and their rarefaction is closely associated with the progression of renal fibrosis (13, 14). Furthermore, we show that caspase-3–dependent apoptosis and autophagy pathways work in concert to regulate microvascular rarefaction and renal fibrosis. Notably, only in the absence of both caspase-3–dependent apoptosis and autophagy that necroptosis becomes the default cell death mechanism in PTCs, with enhanced microvascular rarefaction, renal inflammation, and fibrosis. Collectively, these observations highlight the cell-specific nature of stress and death molecular signaling pathways and demonstrate the predominant role of the renal microvasculature in controlling renal fibrogenesis.

In contrast to the abundance of literature on renal TECs in various models of AKI (23, 26–29), no studies to date have investigated the propensity and dynamics of PCD and autophagy in renal PTCs. In the present study, we show, using publicly available data from the GEO database and validation by PCR in our mouse model, that baseline expression of genes positively regulating apoptosis are enriched in renal endothelial cells. Fas and caspase-8 are important effectors of the extrinsic apoptosis pathway, whereas caspase-12 has been implicated in ER stress–dependent apoptosis and regulation of inflammation (30–32). Consistent with increased propensity toward apoptosis in endothelial cells, we found sustained caspase-3 activation in PTCs during all stages of AKI-to-CKD transition. This pattern differed from that of TECs, where necroptosis was the predominant PCD pathway. PTC necroptosis activation emerged only in *Caspase-3^–/–^* mice treated with CHQ.

Our results add further support to the notion that autophagy and caspase-3 activation within PTCs act as central regulators of microvascular rarefaction and renal fibrogenesis. Although caspase-3 deficiency was associated with enhanced tubular necroptosis in the early stage of AKI, this did not increase renal fibrogenesis, ruling out a major contribution of early tubular injury in renal fibrogenesis. Notably, caspase-3–deficient mice did not exhibit increased PTC necroptosis but instead showed sustained elevations in autophagy indices up to day 21. PTC necroptosis activation emerged only in *Caspase-3^–/–^* mice treated with CHQ, which led to increased leukocyte infiltration, increased PTC rarefaction, and enhanced renal fibrosis. Future studies will aim at characterizing the detailed phenotype of infiltrating cells to better delineate their role in renal fibrogenesis. Collectively, our results identify crosstalk between autophagy and caspase-3–dependent apoptosis in PTCs as a central regulator of microvascular rarefaction and renal fibrosis.

These results add further support to the notion that microvascular rarefaction is a common unifying factor regulating fibrosis in various organs. Enhanced microvascular endothelial apoptosis has been reported in different models of lung fibrosis (33, 34). In patients with non-alcoholic steatohepatitis, impaired autophagy in liver sinusoidal endothelial cells promotes apoptosis and contributes to liver fibrosis (35). Endothelial cell–specific invalidation of ATG-7 was shown to increase development of fibrosis after CCl_4_-induced liver injury (36). Myocardial fibrosis that develops in association with heart failure, either secondary to coronary artery disease, diabetes, or in the context of heart failure with preserved ejection fraction, has also been associated with microvascular rarefaction (37–40). Microvascular rarefaction has been implicated in the development of bladder fibrosis secondary to ischemia (41). Inhibition of autophagy through endothelium-specific deletion of p62 has been associated with microvascular rarefaction as well as heart, lung, and kidney fibrosis (42). Future studies are needed to assess whether crosstalk between caspase-3 and autophagy activation within the microvasculature is also central to regulation of fibrosis in these various disease states.

A number of mechanisms have been implicated in the fibrogenic impact of microvascular rarefaction. It is associated with substantial structural and functional changes (43, 44) that include widening of the subendothelial space, and increased vascular permeability favoring exudation of circulating proteins such as fibrinogen and fibronectin that enhance the stiffness and molecular composition of the ECM (4). This in turn favors differentiation of resident fibroblasts into myofibroblasts characterized by development of stress fibers, expression of ACTA2, and increased collagen production (3, 45). Loss of endothelial integrity and excessive ECM deposition also disrupt endothelial cell–pericyte interactions, leading to pericyte detachment (46) and their subsequent differentiation into a myofibroblast phenotype (47). Notably, experimental depletion of pericytes before injury has been shown to attenuate renal fibrosis (48). Caspase-3–dependent endothelial apoptosis also promotes the production of connective tissue growth factor, which in turn fosters persistent myofibroblast differentiation and fibrosis (49, 50). Thus, caspase-3 activation leading to PTC rarefaction serves as an upstream initiator of various fibrogenic pathways.

Our study has a number of limitations. First, we used CHQ for autophagy disruption instead of genetically deficient murine models of autophagy. However, the vast majority of *Atg*-KO murine models, including *Atg-5*, -*7*, -*12*, and -*16L1*, are either embryonic lethal or associated with early death after birth (51, 52). As our main goal was to study the differences in PCD and autophagy activation in tubules and PTCs are their impact on renal fibrogenesis, we needed to ensure autophagy inhibition in both cell types, ruling out the use of cell-specific *Atg* KO. Second, although we showed that autophagy, apoptosis, and to a lesser degree necroptosis play a role in regulating microvascular rarefaction and renal fibrosis, we have yet to show that endothelial cell–specific caspase-3 invalidation is sufficient to prevent renal fibrogenesis. These studies are under way, as we are currently standardizing endothelial cell–specific caspase-3–KO models. Lastly, other types of PCD, such as ferroptosis and pyroptosis, have been reported in models of AKI (23) and have not been studied in the present work. We chose to focus on caspase-3–dependent apoptosis and p-RIPK3–dependent necroptosis, as both have been reported to play central roles in PTC and TEC PCD during AKI (13, 14, 29, 53). In addition, PCD-induced classical inflammatory mediators, including TNF-α, IL-1β, IL-6, and MCP1, are key regulators of postischemic renal inflammation. However, inflammatory signaling was not directly investigated in this work, and future studies will be required to characterize the importance of other types of PCD on the development of microvascular rarefaction, renal inflammation, and fibrosis.

Overall, our findings provide a detailed side-by-side time course assessment of apoptosis, necroptosis, and autophagy in tubules and PTCs after renal IRI. Our results demonstrate distinct gene expression patterns and death-regulated pathways in TECS and PTCs, with a predominance of necroptosis in tubules and apoptosis in PTCs. Our results also highlight the importance of crosstalk between autophagy and caspase-3–mediated cell death in controlling PTC loss and renal fibrosis. Collectively, these results underscore the need to consider cell specificities, and in particular the central role of the microvasculature, in designing and testing therapeutic strategies aimed at preventing renal fibrosis through modulation of autophagy and regulated death pathways.

## Methods

### Sex as a biological variable.

Our study exclusively examined female mice because male animals had a higher mortality rate. It is unknown whether the findings are relevant for male mice.

### Animal studies.

Eight- to 12-week-old female GFP-LC3 mice (background strain C57BL/6JJcl; stock RBRC00806; RIKEN BioResource Center, Tsukuba, Japan) (54) and caspase-3–deficient mice (*Caspase-3^–/–^*;B6N.129S1-C3^tm1Flv^/J; backcrossed on C57BL/6N; stock 006233; Jackson Laboratory) (55) were used. *Caspase-3^–/–^* mice were crossed with GFP-LC3 mice to generate GFP-LC3/*Caspase-3^–/–^* experimental animals. All comparisons were performed using GFP-LC3/*Caspase-3^+/+^* littermates as controls to ensure a consistent mixed genetic background across all groups. Mice were kept in the animal housing system with 12-hour light-dark cycles and a normal diet ad libitum.

### Renal IRI model.

Renal IRI by unilateral renal pedicle clamping with contralateral nephrectomy was performed as described previously (13, 14, 56). Briefly, mice were anesthetized with 2% isoflurane and 1 L/min oxygen. Carprofen (10 mg/kg) was administered subcutaneously for pain relief. Abdominal region shaved mice were placed on a surgical homeothermic pad. Bupivacaine and lidocaine combo (1:10, 0.25%, 2 mg/kg) were administered subcutaneously on the mid-abdominal incision spot. The left renal pedicle was exposed via mid-abdominal incision, and we clamped the left renal pedicle for 30 minutes. Ischemia induces a change in kidney color from bright red to dark purple and reperfusion by releasing the clamp returns the kidney color to bright red. The right kidney was then exposed, and ligation of the ureter and renal blood vessels with a 4-0 suture was performed before right kidney nephrectomy. Each mouse was examined 2 times per day in the first 2 days after the surgery to check the general condition, body weight, surgical wound, and food. The endpoint was a loss of 20% body weight. GFP-LC3, GFP-LC3/*Caspase-3^+/+^* littermates, and GFP-LC3/*Caspase-3^–/–^* mice were randomly assigned to receive CHQ (Sigma-Aldrich, C6628), which was administered intraperitoneally (100 mg/kg/day) on the day of surgery and every day after surgery until the sacrifice. Mice were sacrificed before surgery or on days 1, 2, 7, and 21 after surgery and the left kidney, serum, and urine were collected from euthanized mice. Only female mice were used for the study because male mice showed severe injury after surgery.

### Biochemical evaluation of renal function.

Serum creatinine levels were measured using Vitro CREA slides and Vitro chemistry products (Ortho Clinical Diagnostics, Vitro 250/350 Chemistry System), as described previously (13, 14). BUN levels were quantified using a QuantiChrom Urea Assay Kit (BioAssay Systems, DIUR-100) following the manufacturer’s instructions.

### Renal endothelial cell isolation.

Mice were anesthetized using 2% isoflurane delivered in 1 L/min oxygen. A 23-gauge catheter (12 cm in length) was inserted into the left ventricle and connected to a syringe filled with 0.9% sodium chloride saline (Baxter, JF7123). The syringe pump (Harvard Apparatus, 33 Syringe Pump) was set to perfuse at a rate of 2.5 mL/min to clear the blood via the right atrium. Harvested kidneys were stored in 8 mL of isolation buffer (DMEM + 20% FBS + 1% Pen/Strep). The immersed tissues were placed on ice until the dissociation and digestion steps. The medium was removed and replaced with 8 mL of digestion solution (0.22-μm filtered 3 mg/mL collagenase I in DMEM). Kidney tissue was shredded using scissors for 2 minutes, and the tube was incubated at 37°C for 60 minutes. The tissue suspension was then passed through a 5 mL syringe fitted with a 20-gauge cannula, and clumps were triturated into a single-cell suspension at least 12 times. The minced tissue was filtered through a 70 μm cell strainer seated atop a 50 mL tube pre-filled with 16.5 mL of isolation solution, which contained serum to stop the digestion. The cell suspension was mixed and centrifuged at 300*g* for 8 minutes at 4°C. The supernatant was carefully removed, and the cell pellet was resuspended with 1 mL of bead washing solution (Dulbecco’s PBS [D-PBS] + 0.1% BSA + 1% Pen/Strep) before being transferred to a 1.5 mL Eppendorf tube. Twenty-five microliters of CD31-coated Dynabeads (BD Pharmingen, 553370) was added to the suspension and incubated at room temperature for 15 minutes with robust shaking on a rocking platform. The cell suspension was placed on a magnetic rack for 1 minute, and the supernatant (containing the CD31^–^ cells) was carefully aspirated. The CD31^+^ cells isolated on magnetic beads were washed 5 times with isolation media and then resuspended in 100 μL lysis buffer to proceed with RNA extraction. In parallel, 200 μL of cell suspension (from the initial postcentrifugation preparation) was transferred to a 1.5 mL Eppendorf tube. Twenty-five microliters of CD31-coated Dynabeads was added to the suspension and incubated at room temperature for another 15 minutes with vigorous shaking on a rocking platform to fully deplete CD31^+^ cells from the cell preparation. The cell suspension was placed on a magnetic rack for 1 minute, and the cell suspension (supernatant) was carefully aspirated to proceed with RNA extraction (CD31^–^ fraction).

### Quantitative RT-PCR.

The expression levels of mRNA were determined using qRT-PCR. Total RNA was isolated from cells using the miRNeasy Tissue Advanced Kit (QIAGEN) according to the manufacturer’s protocol. The total RNA was quantified using a DS-11 Series Spectrophotometer/Fluorometer (DeNovix). Six hundred nanograms of total RNA was treated with RNase-free DNase I (Invitrogen) and reverse transcribed into cDNA using a Reliance Select cDNA Synthesis Kit (Bio-Rad), following the manufacturer’s instructions. qPCR amplification of mRNA was performed using 12 ng of cDNA with the following TaqMan probes from Thermo Fisher Scientific: *Pecam1/CD31* (Mm01242576_m1), *Epcam* (Mm00493214_m1), *Casp8* (Mm01255716_m1), *Casp12* (Mm00438038_m1), *Fas* (Mm01204974_m1), or *Hprt1* (Mm03024075_m1), and TaqMan Fast Advanced Master Mix. The reaction was performed in a total volume of 20 μL. The reaction conditions were as follows: denaturation at 95°C for 20 seconds, followed by 40 cycles of denaturation at 95°C for 1 second and annealing and extension at 60°C for 20 seconds. The fold change in *Casp8*, *Casp12*, and *Fas* mRNA levels was calculated using the comparative Ct method and normalized to *Hrpt1*.

### Kidney processing, histological staining, and immunohistochemistry.

The left kidneys were fixed in 10% formalin, embedded in paraffin, and sliced into 4-μm sections. Immunohistochemical staining was performed on paraffin-embedded tissue using antibodies against cleaved caspase-3 (Biocare Medical, CP229B), p-RIPK3 (Abcam, ab195117), PLVAP (Novus Biologicals, NB100-77668), SQSTM1/p62 (Abcam, ab91526), ACTA2 (Dako/Agilent, IR611), and CD45 (BD Biosciences, 550539). Stained slides were scanned with an Olympus VS110 slide scanner or Leica DM40000B microscope, and randomly chosen fields were evaluated using Olyvia or Aperio ImageScope software.

Quantification of cleaved caspase-3, p-RIPK3, SQSTM1/p62, and ACTA2 staining in PTCs was assessed by evaluating the number of positive PTCs in 5 high-power fields (×200) in the corticomedullary junction and 5 in the cortex. In tubules, we evaluated the ratio of positive tubules to the total number of tubules. Quantification of PLVAP staining was assessed by evaluating the ratio of positive PTCs to tubule number in 5 high-power fields (×200) in the corticomedullary junction and 5 in the cortex. Renal tubular damage was scored on hematoxylin and eosin–stained (H&E-stained) slides. Ten high-power fields (×200) were randomly selected in the renal cortex (5) and corticomedullary junction (5). Quantification of damaged tubules was expressed as tubular injury score (TIS) from 0 to 5, where: 0 (normal); 1 (mild injury, <10%); 2 (moderate injury, 11%–25%); 3 (severe injury, 26%–49%); 4 (high severe injury, 50%–75%); 5 (extensive injury, >75%) (13, 14). PTC microvascular congestion was estimated by counting aggregated erythrocytes inside PTCs in 10 randomly selected high-power fields (×200) in the cortex (5) and corticomedullary junction (5) of H&E-stained slides. Sirius red staining was performed using Direct Red 80 (Sigma-Aldrich, 365548) according to the manufacturer’s protocol. Ten randomly selected high-power fields (×200) in the cortex (5) and corticomedullary junction (5) were captured using Olympus VS110. Sirius red–positive areas, which indicate collagen deposition, were evaluated using ImageJ (NIH). Quantification of CD45 staining was assessed by evaluating the ratio of CD45^+^ area to total tissue area. All assessments were conducted by an independent investigator who was blinded to the experimental conditions, and the results were subsequently validated by a pathologist.

### Confocal microscopy.

Paraffin-embedded sections (4 μm) were deparaffinized in xylene and rehydrated in gradient ethanol (100%, 90%, and 70%). Antigen retrieval was performed by autoclaving the specimens in sodium chloride buffer (pH 9.0) with 0.05% Tween 20 and then blocking them with 1.5% goat serum at room temperature for 30 minutes. The slides were incubated with rat anti-mouse PLVAP (Novus, 100-77668) overnight at 4°C in 1.5% goat serum/D-PBS. Slides were subsequently washed with D-PBS and incubated with Alexa Fluor 647–conjugated goat anti-GFP (Invitrogen, A-31852) and Alexa Fluor 555–conjugated goat anti-rat (Invitrogen, A-21434) in 1.5% goat serum/D-PBS for 30 minutes at room temperature. Slides were washed and incubated with 4,6-diamidino-2-phenylindole (DAPI; Invitrogen, D3571) for 10 minutes and mounted with Prolong Gold antifade reagent mounting medium (Invitrogen, P36934).

High-resolution imaging was performed using an inverted Zeiss Axio Observer 7 LSM 900 Airyscan 2 confocal microscope. Samples were imaged using a Plan-Apochromat 40×/1.3 NA oil immersion objective lens (DIC UV/VIS-IR, M27). Laser scanning confocal microscopy was used for all image acquisitions, employing sequential acquisition in frame scan mode with bidirectional scanning, a mean intensity averaging of 4, and 16-bit depth. The LSM was equipped with an MBS 405 + 488 + 561 + 640 (T10/R90), and all detectors used for imaging were GaAsP-PMTs. In track 1, Alexa Fluor 555 was excited using a diode laser 561 nm, with emissions collected between 564–588 nm. In track 2, DAPI and Alexa Fluor 647 were excited simultaneously using diode lasers at 405 nm and 640 nm, respectively, and emission was collected between 410–470 for DAPI and 656–700 nm for Alexa Fluor 647. For single-plane imaging, images were acquired with a pixel size of 0.076 μm × 0.076 μm, a resolution of 2101 × 2101 pixels, and a scanning speed of 1.00 μs/pixel. For *Z*-stack imaging, images were acquired with a zoom of 2.0×, a pixel size of 0.076 μm × 0.076 μm, a scanning speed of 2.01 μs/pixel, and a *Z*-step size of 0.25 μm, resulting in 30–40 slices per stack with a resolution of 1051 × 1051 pixels per plane. For track 1, imaging was performed with a pinhole size of 1 AU, while track 2 used a pinhole size of 1.06 AU, ensuring optimal sectioning with a *Z*-stack step size of 0.25 μm. Images were acquired using ZEN (Blue Edition) software (version 3.6.095.04000) and subsequently processed in ImageJ.

PLVAP^+^ cells (stained with Alexa Fluor 555) were considered as PTCs, while autofluorescence (green) was used to identify tubules (57–59). Autophagic flux (stained with Alexa Fluor 647) was manually counted.

### Electron microscopy.

Mouse kidneys were collected and fixed in 3% glutaraldehyde (Avantor, 100503-990) for at least 24 hours at 4°C. The samples were then processed using a tissue processor (Leica EM TP), which included secondary fixation with osmium tetroxide (OsO_4_, osmium acid) solution, followed by dehydration and embedding in EPON resin according to the protocol established by the Centre Hospitalier Universitaire Sainte-Justine (CHU Sainte-Justine) pathology platform. Semi-thin sections were prepared for tissue evaluation, and ultra-thin sections were subsequently obtained using an ultramicrotome (Leica EM UC7) and mounted on nickel grids. The sections were stained with aqueous uranyl acetate and lead citrate. Imaging was performed using a transmission electron microscope (Talos L120C) equipped with a Ceta CMOS camera (4K × 4K) (60).

### Ex vivo renal microvascular microCT imaging.

The following protocol was adapted from Lan et al. (14). Mice were anesthetized using 2% isoflurane and 1% oxygen. A catheter (12 cm in length, 23 gauge) was inserted into the left ventricle and connected to a syringe filled with 0.9% sodium chloride saline (Baxter, JF7123). The syringe pump (Harvard Apparatus, 33 Syringe Pump) was set to perfuse at a rate of 2.5 mL/min to remove blood via the right atrium, followed by fixation with 10% buffered formalin (ChapTec, TUY4010). Afterward, a second perfusion with 0.9% sodium chloride saline was performed and the radiopaque contrast agent Microfil (Flow tech, LMV-122) was introduced to allow 3D visualization of the renal microvasculature. The kidneys were collected following polymerization for 1 hour at room temperature and stored in a 1.5 mL tube containing 10% buffered formalin for subsequent scanning procedures. Ex vivo microCT manipulation was conducted using a high-resolution SkyScan 1176 scanner (Bruker). The fixed kidney was positioned and scanned 180° around the vertical axis with a rotation increment of 0.48°, with a resolution of 9 μm. Vascular volume was evaluated through volume rendering and 3D reconstruction analysis while terminal capillaries were counted using Imaris 9.7.2 software (Oxford Instruments).

### Statistics.

All data are presented as the mean ± SEM of at least 3 independent experiments unless otherwise indicated. For animal experiments, the sample size indicated as “*n*” in the figure legends represents individual kidneys, with each replicate corresponding to a kidney collected from a distinct mouse. Data were compared by 2-tailed Student’s *t* test, 1-way ANOVA, or 2-way ANOVA with Prism (GraphPad Software Inc.). A *P* value of less than 0.05 was considered significant.

### Study approval.

All animal experimental protocols were reviewed and approved by the Center Hospitalier de l’Université de Montréal-Comitée Institutionnel de Protection des Animaux (CIPA).

### Data availability.

Values for all data points in graphs are reported in the Supporting Data Values file.

## Author contributions

HK, FM, JT, M Dieudé, and MJH conceived and designed the research program. HK, SL, IK, AKR, SQ, and M Dupont performed the experiments. SQ provided technical and experimental support for the in vivo mouse renal IRI model. HK, FM, and IK analyzed the data. HK, FM, IK, JT, M Dieudé, and MJH interpreted the results. HK and FM prepared the figures. HK, FM, JT, and MJH drafted the manuscript. HK, FM, JT, and MJH edited and revised the manuscript. All the authors approved the final version of the manuscript.

## Conflict of interest

The authors have declared that no conflict of interest exists.

## Funding support

- Canadian Donation and Transplantation Research Program.
- Canadian Institutes of Health Research grant PJT-155985 (to MJH).
- Canadian Society of Transplant Research Training Award (to HK).
- CHUM Foundation Immunopathology Scholarship (to HK).
- J.-L. Lévesque Foundation (to MJH).
- Canadian Foundation of Innovation (CFI) grant 42649 (to CRCHUM).
- Shire Chair in Nephrology, Transplantation, and Renal Regeneration of the Université de Montréal (to MJH).

## Supplementary Material

Supplemental data

Unedited blot and gel images

Supporting data values

## Version 1 07/08/2026

Electronic publication
