# Supplementary material for: Microvascular autophagy and caspase-3 activation are central regulators of renal fibrosis after ischemia-reperfusion
Source: JCI Insight. 2026 Jul 8;11(13):e201945. doi: 10.1172/jci.insight.201945 (PMC13460856; doi:10.1172/jci.insight.201945)
Supplement: Supplementary file 1 [file jciinsight-11-201945-s092.pdf]

Supplemental material

Kidney tissue lysis, protein isolation, and immunoblotting

The following protocol was adapted from Yang et al. [1]. Kidney tissues were snap frozen in liquid nitrogen and stored at -80°C immediately on the mice sacrifice day. Tissue samples were homogenized manually in RIPA lysis buffer containing phosphatase inhibitor (Calbiochem, 524625), protease inhibitor (Sigma, P1860), and SDS 10%. The protein concentration was measured by BCA assay (Pierce, 23225). Protein (20 ug) from lysates was loaded on a 10%, 12% and 15% polyacrylamide gel and separated by SDS-PAGE. Proteins were transferred to nitrocellulose membranes (Bio-Rad, 162-0115), blocked in 5% skimmed milk in PBS containing 0.1% Tween (PBST), and probed overnight at 4°C with the following primary antibodies: rabbit anti-LC3 at 1:1000 (Novus, NB600-1384); rabbit anti-SQSTM1/p62 at 1:2000(Abcam, ab91526); goat anti-TIM-1/KIM-1/HAVCR at 1:1000 (Novus Biologicals, AF1817); rabbit anti-α1β tubulin at 1:2000 (Proteintech, 11-224-1-AP); rabbit anti-GFP at 1:1000 (Cell signaling, 2956S). Following incubation with primary antibodies, the blots were washed, probed with the Anti-Rabbit IgG HRP at 1:8000 (Abcam, ab7074) or Anti-Goat IgG HRP at 1:10000 (R&D system, HAF017) for 30 min at room temperature, and then visualized using the western ECL kit (Bio-Rad, 1705062). Quantification of western blot bands was performed using Image Lab software (Bio-Rad).

Supplementary Figure Legends

Figure S1. Renal ischemia-reperfusion injury induces kidney dysfunction and tubular damage. (**A**) Bar graph of serum creatinine levels in GFP-LC3 mice post-IRI (n=3-5). (**B**) Bar graph of blood urea nitrogen levels in GFP-LC3 mice post-IRI (n=3-5). (**C**) Bar graph of mean tubular injury scores in H&E-stained kidney sections (n=4-5). (**D**) Bar graph of mean number of PTC congestion in H&E-stained kidney sections (n=4-5). Quantification was performed with samples at baseline, 1, 2, 7, and 21 days post-IRI. For each kidney, ten randomly selected high-power fields (200× original magnification) were evaluated, consisting of five fields from the cortex and five from the corticomedullary junction. Values are mean ± SEM. P values obtained by one-way ANOVA and the Bonferroni post hoc test. *$p<0.05$, **$p<0.01$, ***$p<0.001$, and ****$p<0.0001$, compared between baseline and each time points.

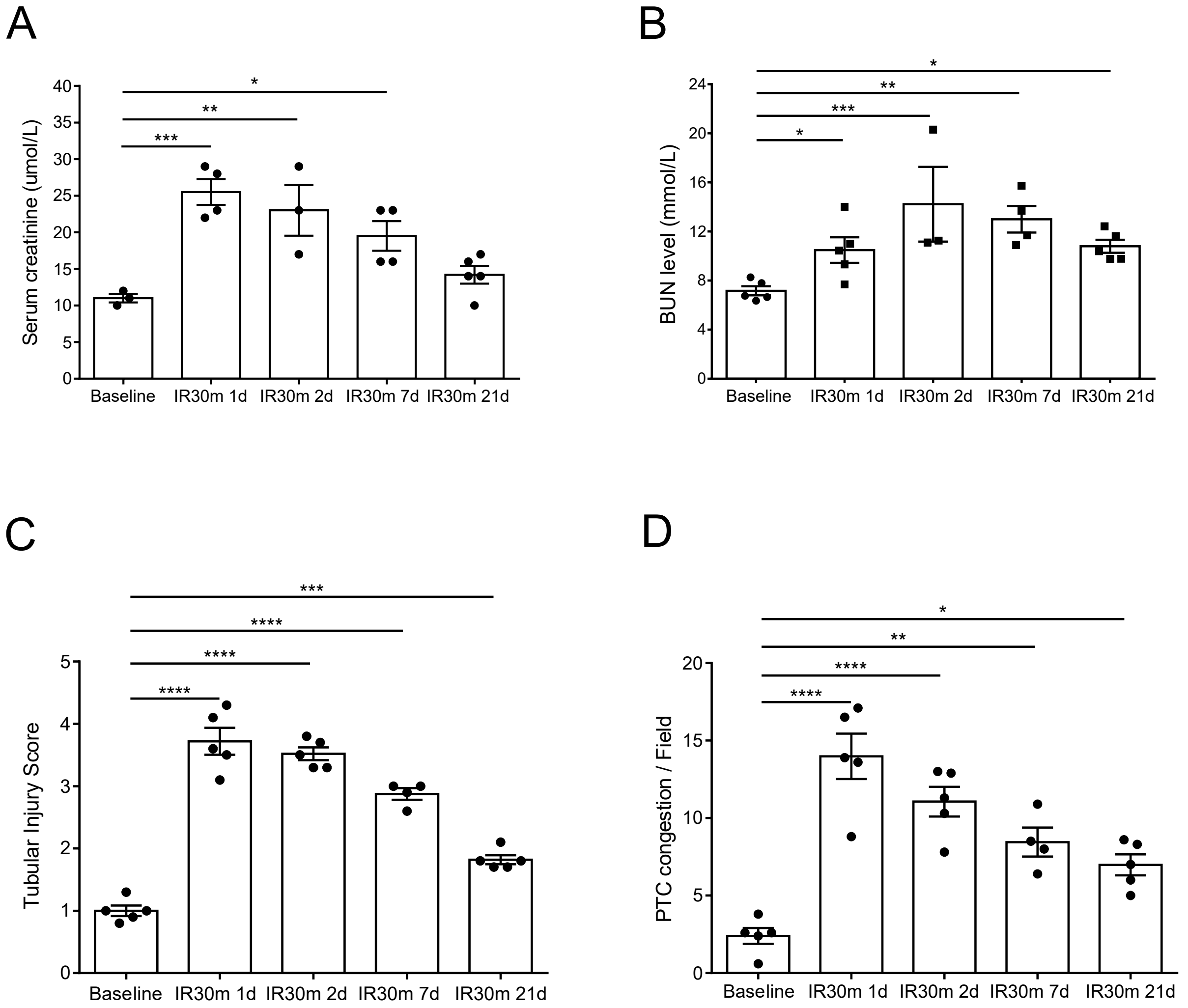
A
Serum creatinine (umol/L)
0
5
10
15
20
25
30
35
40
***
**
*
Baseline
IR30m 1d
IR30m 2d
IR30m 7d
IR30m 21d
B
BUN level (mmol/L)
0
4
8
12
16
20
24
*
***
**
*
Baseline
IR30m 1d
IR30m 2d
IR30m 7d
IR30m 21d
C
Tubular Injury Score
0
1
2
3
4
5
****
****
****
***
Baseline
IR30m 1d
IR30m 2d
IR30m 7d
IR30m 21d
D
PTC congestion / Field
0
5
10
15
20
****
****
**
*
Baseline
IR30m 1d
IR30m 2d
IR30m 7d
IR30m 21d

Figure S2. Renal ischemia-reperfusion injury induces unique patterns of necroptosis and caspase-3-dependent apoptosis in tubules and peritubular capillaries. (**A**) Bar graph of cleaved caspase-3 IHC quantification in renal tubules (n=4-5). (**B**) Bar graph of pRIPK3 IHC quantification in renal tubules (n=3-5). (**C**) Bar graph of cleaved caspase-3 IHC quantification in renal PTC (n=4-5). (**D**) Bar graph of pRIPK3 IHC quantification in renal PTC (n=3-5). Quantification was performed with samples at baseline and 1, 2, 7, and 21 days post-IRI. For each kidney, ten randomly selected high-power fields (200× original magnification) were evaluated, consisting of five fields from the cortex and five from the corticomedullary junction. Values are mean ± standard error of the means (SEM). P values obtained by one-way ANOVA and the Bonferroni post hoc test. $^{**}p<0.01$, $^{***}p<0.001$, and $^{****}p<0.0001$, compared between baseline and each time point.

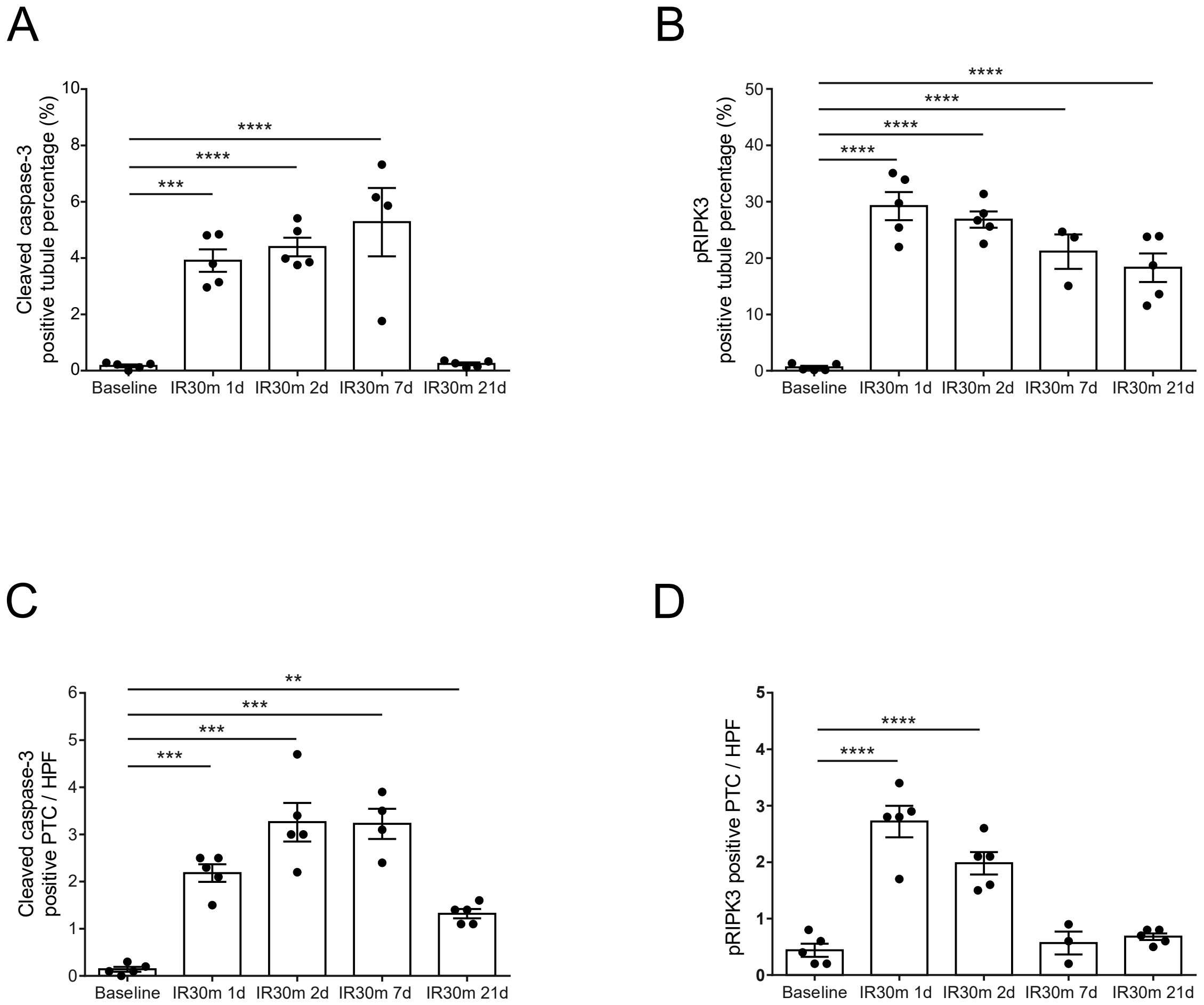
A
Cleaved caspase-3
positive tubule percentage (%)
10
8
6
4
2
0
****
****
***
Baseline
IR30m 1d
IR30m 2d
IR30m 7d
IR30m 21d
B
pRIPK3
positive tubule percentage (%)
50
40
30
20
10
0
****
****
****
****
Baseline
IR30m 1d
IR30m 2d
IR30m 7d
IR30m 21d
C
Cleaved caspase-3
positive PTC / HPF
6
5
4
3
2
1
0
**
***
***
***
Baseline
IR30m 1d
IR30m 2d
IR30m 7d
IR30m 21d
D
pRIPK3 positive PTC / HPF
5
4
3
2
1
0
****
****
Baseline
IR30m 1d
IR30m 2d
IR30m 7d
IR30m 21d

Figure S3. Autophagy dynamics in tubules and peritubular capillaries following renal ischemia-reperfusion injury. (**A**) Bar graph of GFP-LC3 puncta quantification in renal tubules (n=5). (**B**) Bar graph of SQSTM1/p62 IHC quantification in renal tubules (n=4-5). (**C**) Bar graph of autophagic puncta quantification in renal PTC (n=3-5). (**D**) Bar graph of SQSTM1/p62 IHC quantification in renal PTC (n=4-5). All quantification was performed with samples at baseline, 1, 2, 7, and 21 days post-IRI. For each kidney, ten randomly selected high-power fields (200× original magnification) were evaluated, consisting of five fields from the cortex and five from the corticomedullary junction. Values are mean ± standard error of the means (SEM). P values obtained by one-way ANOVA and the Bonferroni post hoc test. **p<0.01, ***p<0.001, and ****p<0.0001, compared between baseline and each time point.

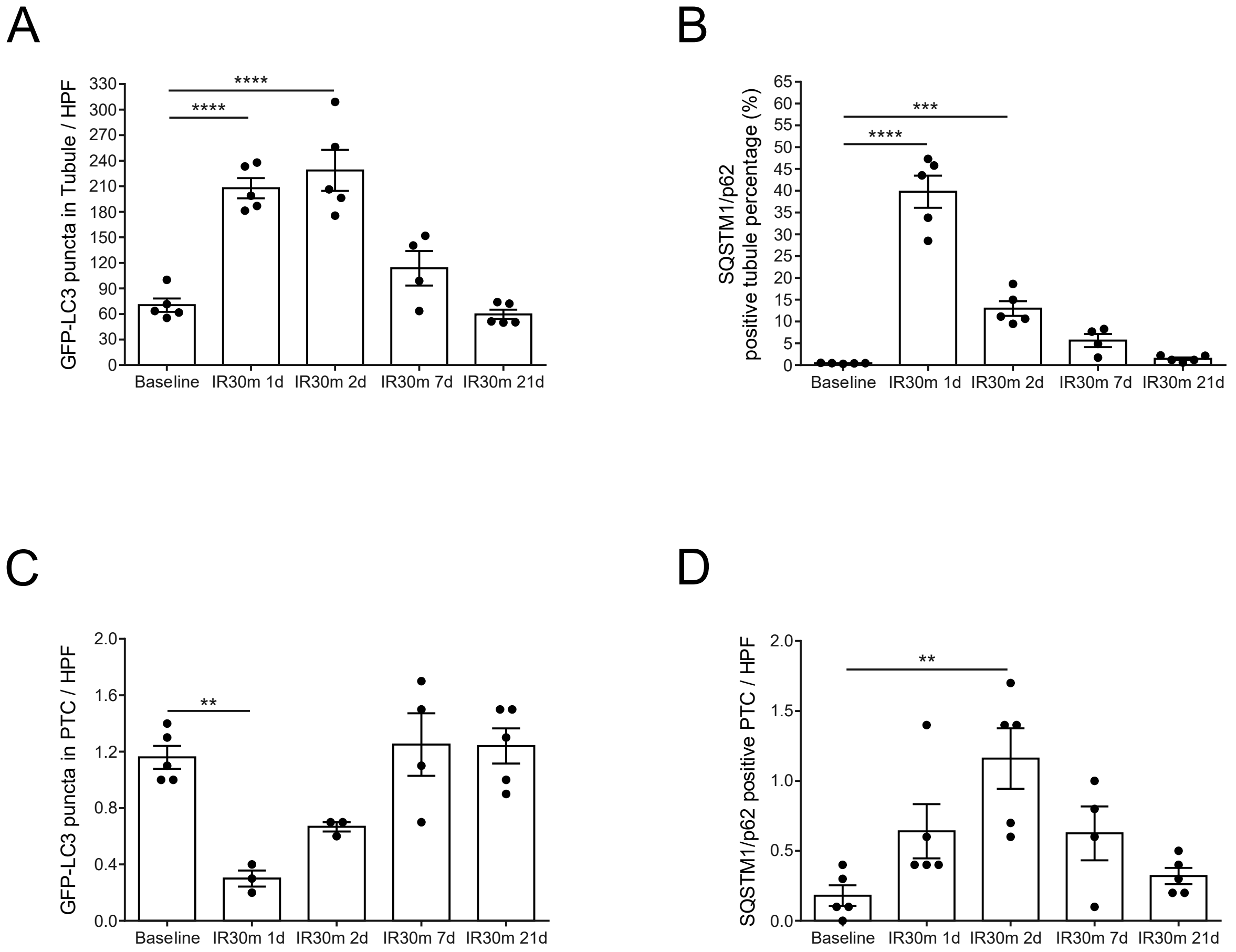
A
B
C
D
GFP-LC3 puncta in Tubule / HPF
SQSTM1/p62
positive tubule percentage (%)
GFP-LC3 puncta in PTC / HPF
SQSTM1/p62 positive PTC / HPF
****
***
**
Baseline
IR30m 1d
IR30m 2d
IR30m 7d
IR30m 21d

Figure S4. Western blot of autophagy-related proteins in GFP-LC3 mice kidney following renal ischemia-reperfusion injury. (**A**) Representative western blots of autophagy markers SQSTM1/p62, LC3-I, LC3-II, and GFP fragment and tubulin in GFP-LC3 mouse kidney. (**B**)–(**D**) Western blot quantification of SQSTM1/p62 (**B**), GFP fragment (**C**), LC3-II/LC3-I (**D**). Quantification was compared between 1-day post-IRI and each time point (n=5). Values are mean ± standard deviation (SD). P values obtained by one-way ANOVA and the Bonferroni post hoc test. *$p<0.05$, **$p<0.01$, and ***$p<0.001$ compared between 1-day post-IRI and each time point.

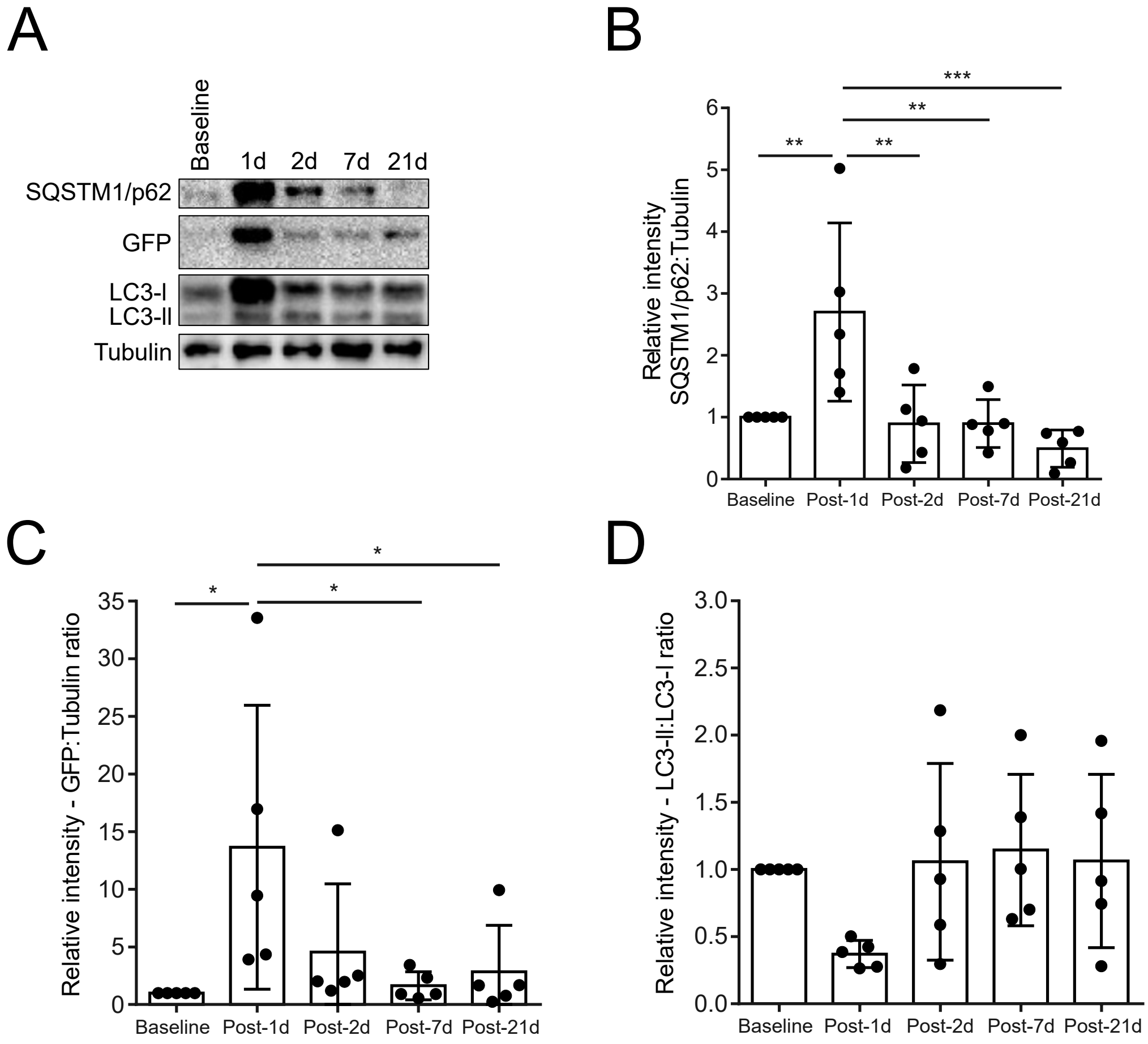

A
Baseline
1d
2d
7d
21d
SQSTM1/p62
GFP
LC3-I
LC3-II
Tubulin
B
Relative intensity
SQSTM1/p62:Tubulin
Baseline
Post-1d
Post-2d
Post-7d
Post-21d
C
Relative intensity - GFP:Tubulin ratio
Baseline
Post-1d
Post-2d
Post-7d
Post-21d
D
Relative intensity - LC3-II:LC3-I ratio
Baseline
Post-1d
Post-2d
Post-7d
Post-21d

Figure S5. Autophagy inhibition enhances kidney dysfunction following renal ischemia-reperfusion injury. Serum creatinine levels up to 21 days with PBS or CHQ post renal-IRI (n=5-8). Quantification was performed by comparing the PBS and CHQ groups at baseline, 1, 2, 7, and 21 days post-IRI. Values are mean ± standard error of the means (SEM). P values obtained by one-way ANOVA and the Bonferroni post hoc test. ***$p<0.001$.

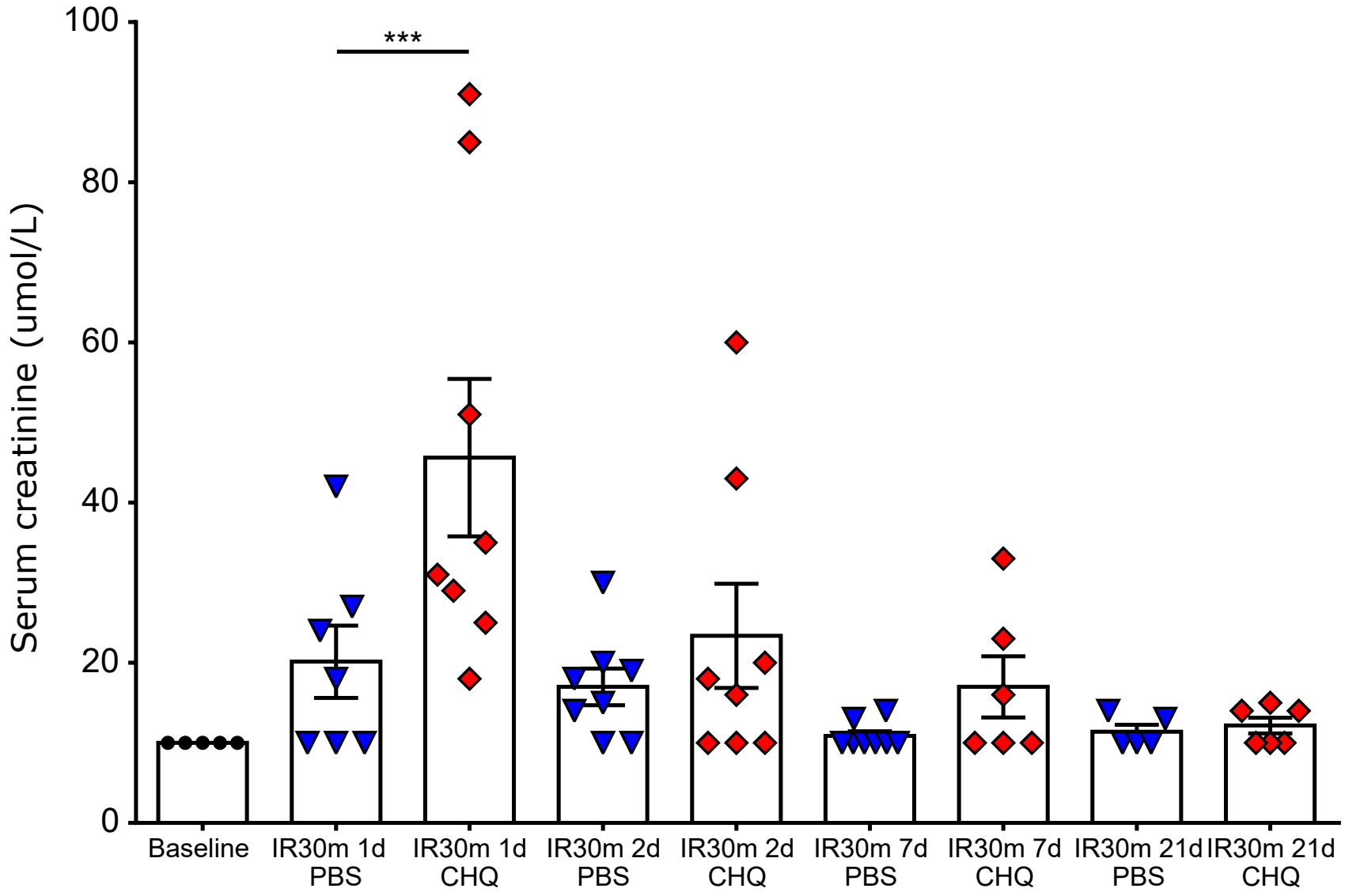
Serum creatinine (umol/L)
100
80
60
40
20
0
***
Baseline
IR30m 1d PBS
IR30m 1d CHQ
IR30m 2d PBS
IR30m 2d CHQ
IR30m 7d PBS
IR30m 7d CHQ
IR30m 21d PBS
IR30m 21d CHQ

Figure S6. Autophagy inhibition enhances autophagosome formation following renal ischemia-reperfusion injury. (**A**) Bar graph of GFP-LC3 puncta quantification in renal tubules from PBS and CHQ-injected mice (n=4-8). (**B**) Bar graph of autophagic puncta quantification in renal PTC from PBS and CHQ-injected mice (n=4-8). Quantification was performed with samples at baseline and 1, 2, 7, and 21 days post-IRI. For each kidney, ten randomly selected high-power fields (200× original magnification) were evaluated, consisting of five fields from the cortex and five from the corticomedullary junction. Values are mean ± standard error of the means (SEM). P values obtained by one-way ANOVA and the Bonferroni post hoc test. *$p<0.05$ and ****$p<0.0001$, compared between PBS and CHQ at the same time points.

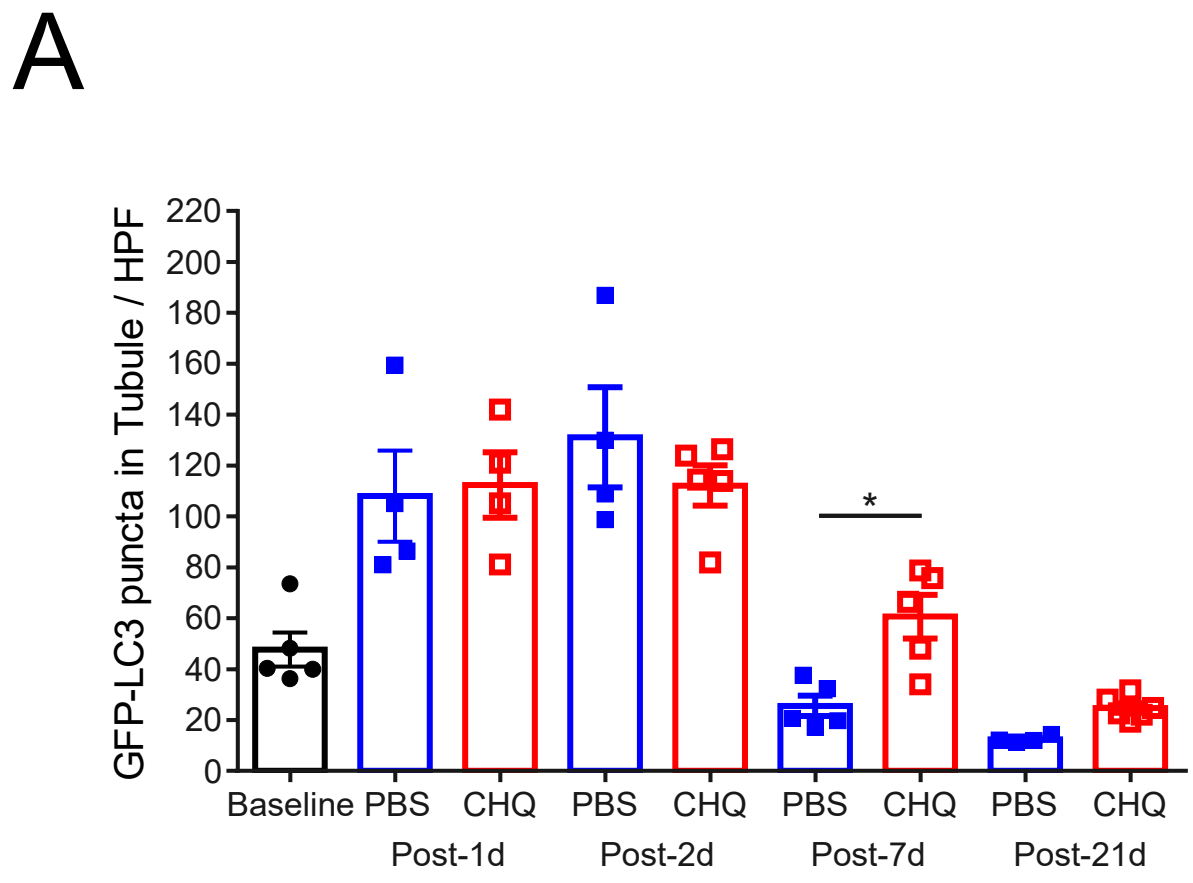
A
GFP-LC3 puncta in Tubule / HPF
220
200
180
160
140
120
100
80
60
40
20
0
*
Baseline
PBS
CHQ
PBS
CHQ
PBS
CHQ
PBS
CHQ
Post-1d
Post-2d
Post-7d
Post-21d

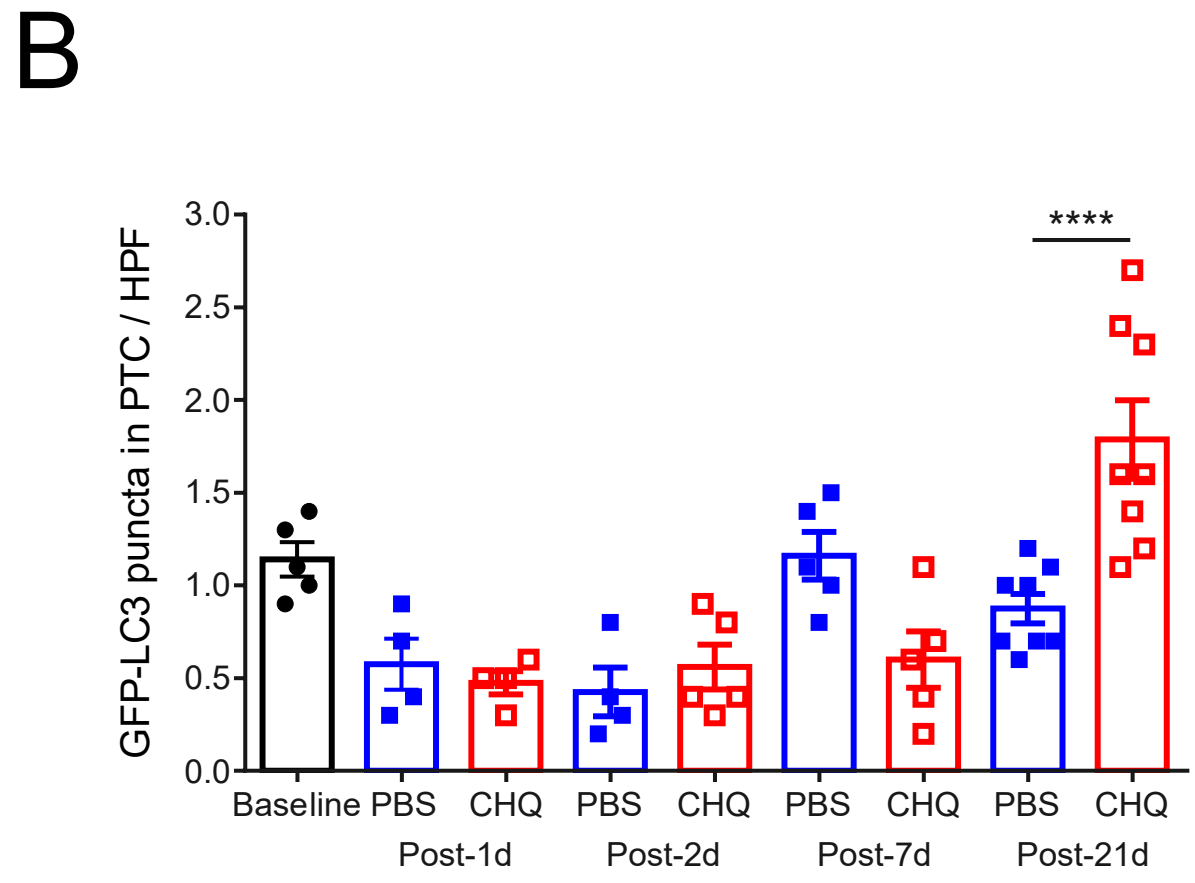
B
GFP-LC3 puncta in PTC / HPF
3.0
2.5
2.0
1.5
1.0
0.5
0.0
****
Baseline
PBS
CHQ
PBS
CHQ
PBS
CHQ
PBS
CHQ
Post-1d
Post-2d
Post-7d
Post-21d

Figure S7. Autophagy inhibition enhances peritubular capillary apoptosis but not tubular necroptosis following renal ischemia-reperfusion injury. (**A**) Bar graph of cleaved caspase-3 IHC quantification in renal tubules from PBS and CHQ-injected mice (n=4-5). (**B**) Bar graph of pRIPK3 IHC quantification in renal tubules from PBS and CHQ-injected mice (n=4-5). (**C**) Bar graph of cleaved caspase-3 IHC quantification in renal PTC from PBS and CHQ-injected mice (n=4-5). (**D**) Bar graph of pRIPK3 IHC quantification in renal PTC from PBS and CHQ-injected mice (n=4-5). Quantifications was performed with samples at baseline and 1, 2, 7, and 21 days post-IRI. For each kidney, ten randomly selected high-power fields (200× original magnification) were evaluated, consisting of five fields from the cortex and five from the corticomedullary junction. Values are mean ± standard error of the means (SEM). P values obtained by one-way ANOVA and the Bonferroni post hoc test. *p<0.05 and **p<0.01, compared between PBS and CHQ at the same time points.

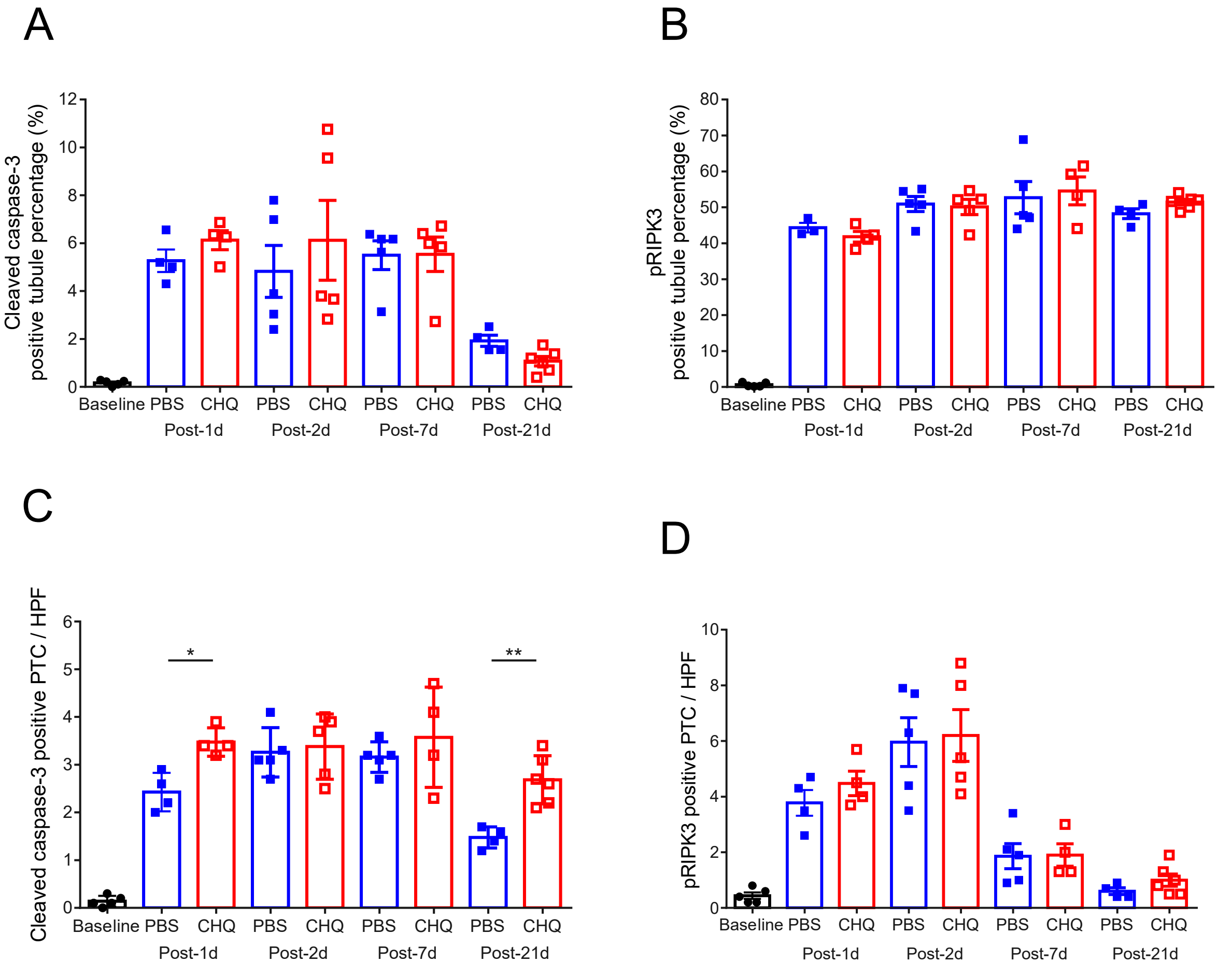
A
Cleaved caspase-3 positive tubule percentage (%)
0
2
4
6
8
10
12
Baseline
PBS
CHQ
PBS
CHQ
PBS
CHQ
PBS
CHQ
Post-1d
Post-2d
Post-7d
Post-21d
B
pRIPK3 positive tubule percentage (%)
0
10
20
30
40
50
60
70
80
Baseline
PBS
CHQ
PBS
CHQ
PBS
CHQ
PBS
CHQ
Post-1d
Post-2d
Post-7d
Post-21d
C
Cleaved caspase-3 positive PTC / HPF
0
1
2
3
4
5
6
*
**
Baseline
PBS
CHQ
PBS
CHQ
PBS
CHQ
PBS
CHQ
Post-1d
Post-2d
Post-7d
Post-21d
D
pRIPK3 positive PTC / HPF
0
2
4
6
8
10
Baseline
PBS
CHQ
PBS
CHQ
PBS
CHQ
PBS
CHQ
Post-1d
Post-2d
Post-7d
Post-21d

Figure S8. Caspase-3 deficiency reduces fibrosis and improves microvascular density following renal ischemia-reperfusion injury. (**A**) Quantification of alpha smooth muscle actin (ACTA2) positive peritubular capillary (PTC) immunohistochemistry in the kidney from GFP-LC3/Caspase-$3^{-/-}$ and GFP-LC3/Caspase-$3^{+/+}$ at 21 days post-IRI (n=5). (**B**) Quantification of Sirius Red staining in the kidney from GFP-LC3/Caspase-$3^{-/-}$ and GFP-LC3/Caspase-$3^{+/+}$ at 21 days post-IRI (n=5). (**C**) Quantification of PLVAP IHC in the kidney from GFP-LC3/Caspase-$3^{-/-}$ and GFP-LC3/Caspase-$3^{+/+}$ at 21 days post-IRI (n=5). For each kidney, ten randomly selected high-power fields (200× original magnification) were evaluated, consisting of five fields from the cortex and five from the corticomedullary junction. Values are mean ± standard error of the means (SEM). P values obtained by unpaired t- test. *p<0.05 and **p<0.01, ***p<0.001 compared between GFP-LC3/Caspase-$3^{-/-}$ and GFP-LC3/Caspase-$3^{+/+}$ at 21 days post-IRI.

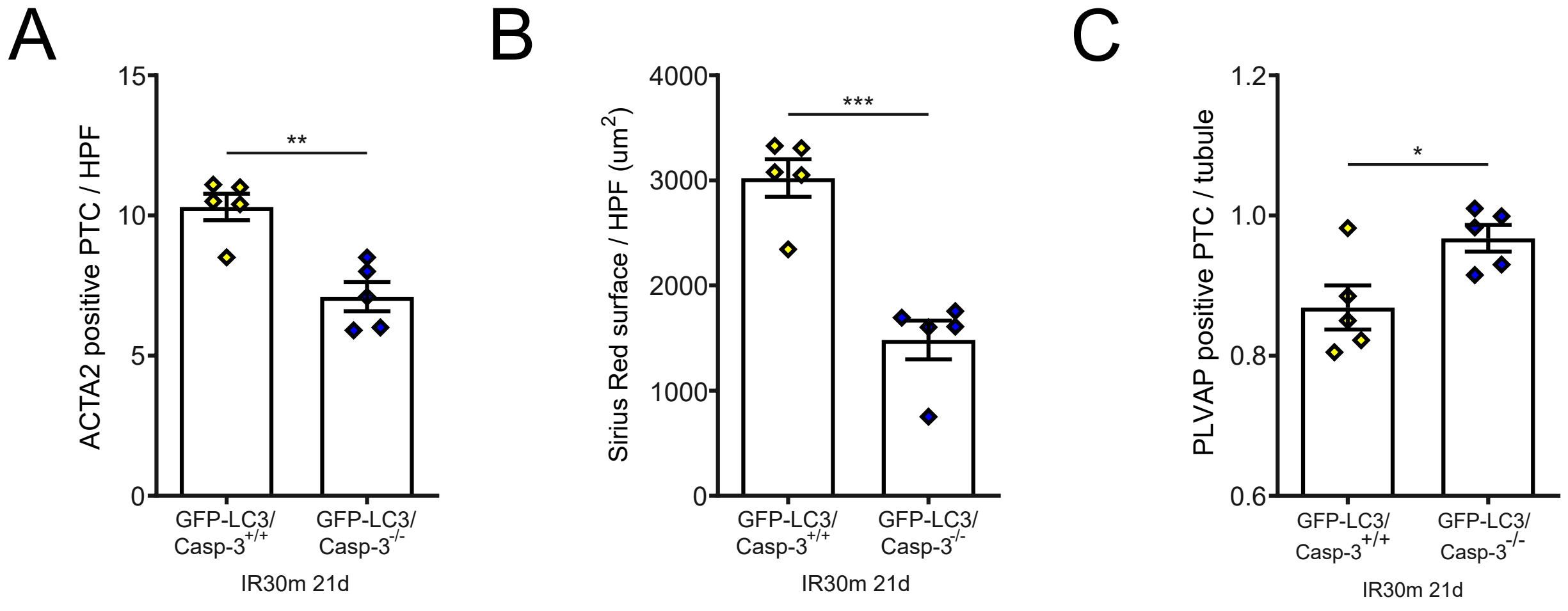
A
ACTA2 positive PTC / HPF
15
10
5
0
**
GFP-LC3/
Casp-3+/+
GFP-LC3/
Casp-3-/-
IR30m 21d
B
Sirius Red surface / HPF (um2)
4000
3000
2000
1000
0
***
GFP-LC3/
Casp-3+/+
GFP-LC3/
Casp-3-/-
IR30m 21d
C
PLVAP positive PTC / tubule
1.2
1.0
0.8
0.6
*
GFP-LC3/
Casp-3+/+
GFP-LC3/
Casp-3-/-
IR30m 21d

Figure S9. Autophagy disruption and caspase-3 deficiency do not affect renal dysfunction and kidney injury following renal ischemia-reperfusion injury. (**A**) Serum creatinine levels at baseline, 2 days, and 21 days with PBS or CHQ post renal-IRI (n=4-5). (**B**) Representative immunoblots of kidney lysate following renal IRI. (**C**) Quantification of immunoblots (n=5). Values are mean ± standard error of the means (SEM). P values obtained by one-way ANOVA and the Bonferroni post hoc test. *p<0.05 and **p<0.01.

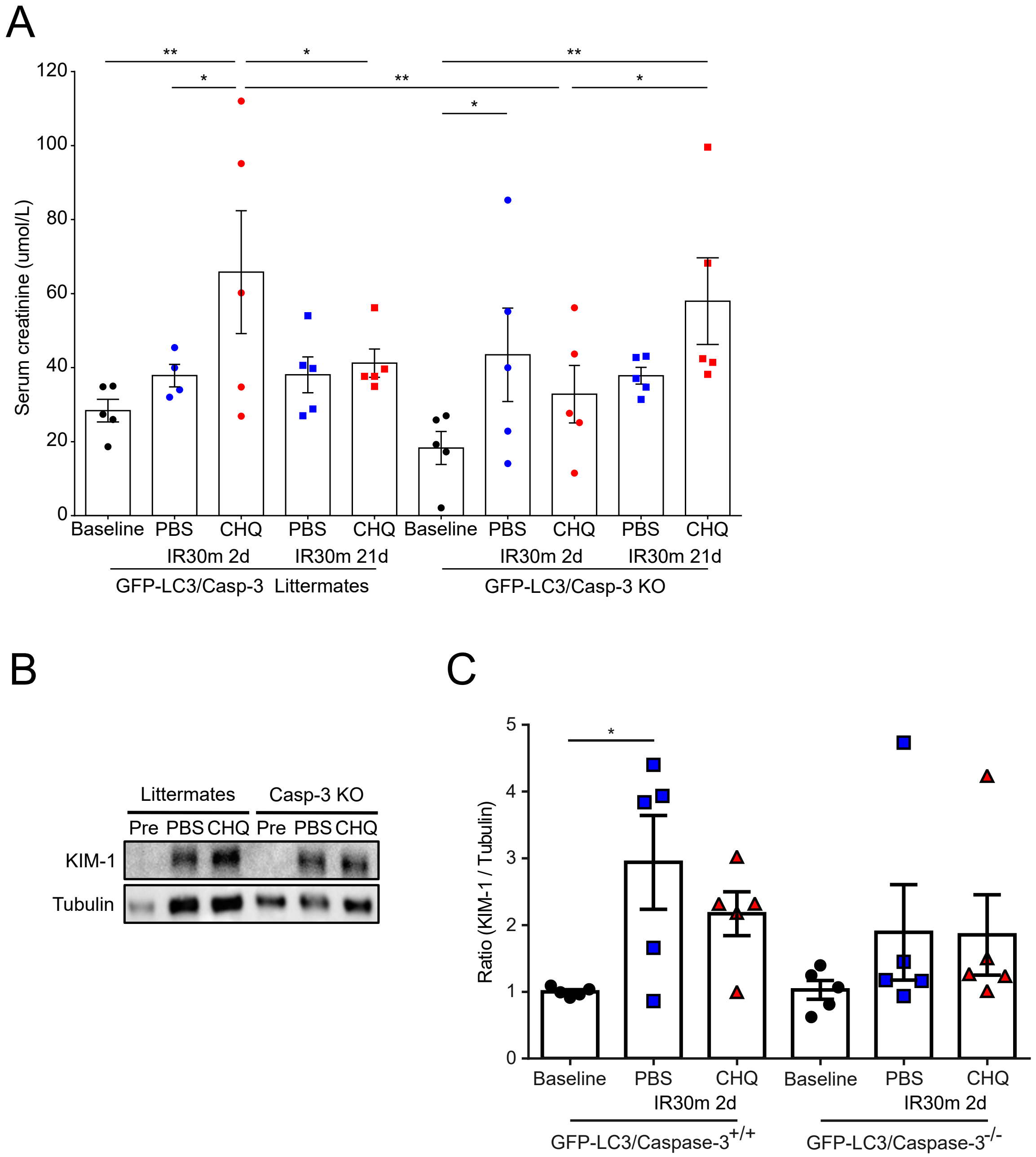

A
Serum creatinine (umol/L)
120
100
80
60
40
20
0
**
*
*
**
*
**
*
Baseline
PBS
CHQ
PBS
CHQ
Baseline
PBS
CHQ
PBS
CHQ
IR30m 2d
IR30m 21d
IR30m 2d
IR30m 21d
GFP-LC3/Casp-3 Littermates
GFP-LC3/Casp-3 KO
B
Littermates
Casp-3 KO
Pre
PBS
CHQ
Pre
PBS
CHQ
KIM-1
Tubulin
C
Ratio (KIM-1 / Tubulin)
5
4
3
2
1
0
*
Baseline
PBS
CHQ
Baseline
PBS
CHQ
IR30m 2d
IR30m 2d
GFP-LC3/Caspase-3+/+
GFP-LC3/Caspase-3-/-

Figure S10. Autophagy disruption and caspase-3 deficiency worsen microvascular rarefaction, fibrosis, and leukocyte infiltration following renal ischemia-reperfusion injury. (**A**) Bar graph of autophagic puncta quantification in renal PTC at 21 days post-IRI from GFP-LC3/Caspase-3$^{-/-}$ and GFP-LC3/Caspase-3$^{+/+}$ injected with PBS or chloroquine (CHQ) (n=5-7). (**B**) Bar graph of SQSTM1/p62 quantification in renal PTC at 21 days post-IRI from GFP-LC3/Caspase-3$^{-/-}$ and GFP-LC3/Caspase-3$^{+/+}$ injected with PBS or chloroquine (CHQ) (n=5-7). (**C**) Bar graph of cleaved caspase-3 IHC quantification in PTC at 21 days post-IRI from GFP-LC3/Caspase-3$^{-/-}$ and GFP-LC3/Caspase-3$^{+/+}$ injected with PBS or chloroquine (CHQ) (n=5-7). (**D**) Relative change in phosphorylated RIPK3 (pRIPK3) IHC in PTC at 2 days post-IRI (n=5). (**E**) Bar graph of pRIPK3 IHC quantification in PTC at 2 days post-IRI from GFP-LC3/Caspase-3$^{-/-}$ and GFP-LC3/Caspase-3$^{+/+}$ injected with PBS or chloroquine (CHQ) (n=4-5). (**F**) Bar graph of pRIPK3 IHC quantification in PTC at 21 days post-IRI from GFP-LC3/Caspase-3$^{-/-}$ and GFP-LC3/Caspase-3$^{+/+}$ injected with PBS or chloroquine (CHQ) (n=5-7). (**G**) Bar graph of PLVAP quantification in renal PTC at 21 days post-IRI from GFP-LC3/Caspase-3$^{-/-}$ and GFP-LC3/Caspase-3$^{+/+}$ injected with PBS or chloroquine (CHQ) (n=5-7). (**H**) Bar graph of ACTA2 quantification in renal PTC at 21 days post-IRI from GFP-LC3/Caspase-3$^{-/-}$ and GFP-LC3/Caspase-3$^{+/+}$ injected with PBS or chloroquine (CHQ) (n=5-7). (**I**) Relative change in Sirius red staining in kidney at 21 days post-IRI from GFP-LC3/Caspase-3$^{-/-}$ and GFP-LC3/Caspase-3$^{+/+}$ injected with PBS or chloroquine (CHQ) (n=5-7). (**J**) Bar graph of Sirius red staining in kidney at 21 days post-IRI from GFP-LC3/Caspase-3$^{-/-}$ and GFP-LC3/Caspase-3$^{+/+}$ injected with PBS or chloroquine (CHQ) (n=5-7). (**K**) Bar graph of CD45-positive area to total tissue area in kidney at 21 days post-IRI from GFP-LC3/Caspase-3$^{-/-}$ and GFP-LC3/Caspase-3$^{+/+}$ injected with PBS or chloroquine (CHQ) (n=5-7). Randomly selected ten high-power fields images (Original

magnification X200) were evaluated from GFP-LC3/caspase-$3^{-/-}$ and GFP-LC3/Caspase-$3^{+/+}$ kidneys from mice injected with PBS or CHQ at 2 and 21 days post-IRI. For each kidney, ten randomly selected high-power fields (200× original magnification) were evaluated, consisting of five fields from the cortex and five from the corticomedullary junction. Values are mean ± SEM. P values obtained by one-way ANOVA and the Bonferroni post hoc test. *p<0.05, **p<0.01, ***p<0.001, and ****p<0.0001.

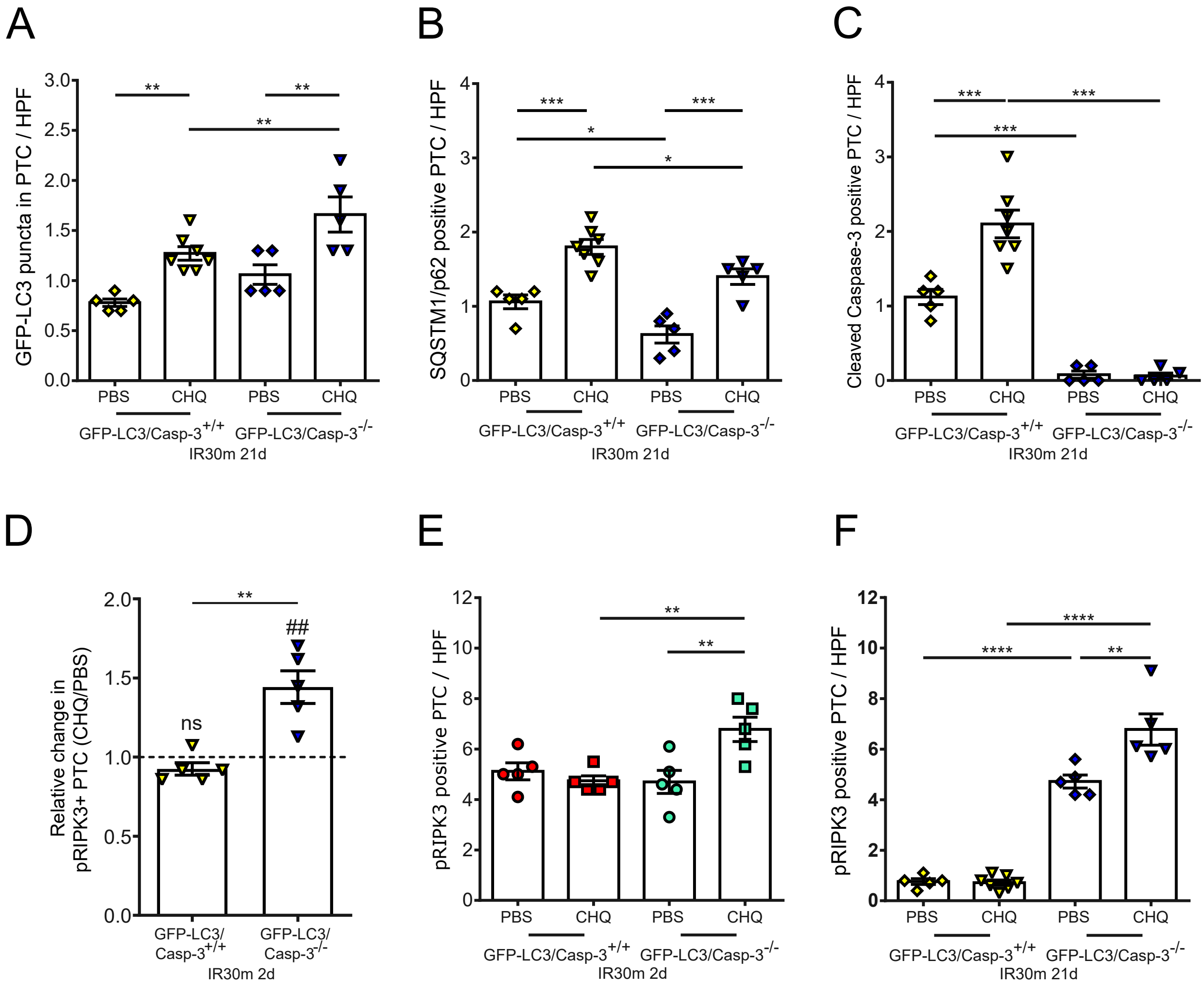
A
GFP-LC3 puncta in PTC / HPF
PBS
CHQ
GFP-LC3/Casp-3+/+
GFP-LC3/Casp-3-/-
IR30m 21d
B
SQSTM1/p62 positive PTC / HPF
C
Cleaved Caspase-3 positive PTC / HPF
D
Relative change in pRIPK3+ PTC (CHQ/PBS)
ns
##
IR30m 2d
E
pRIPK3 positive PTC / HPF
F

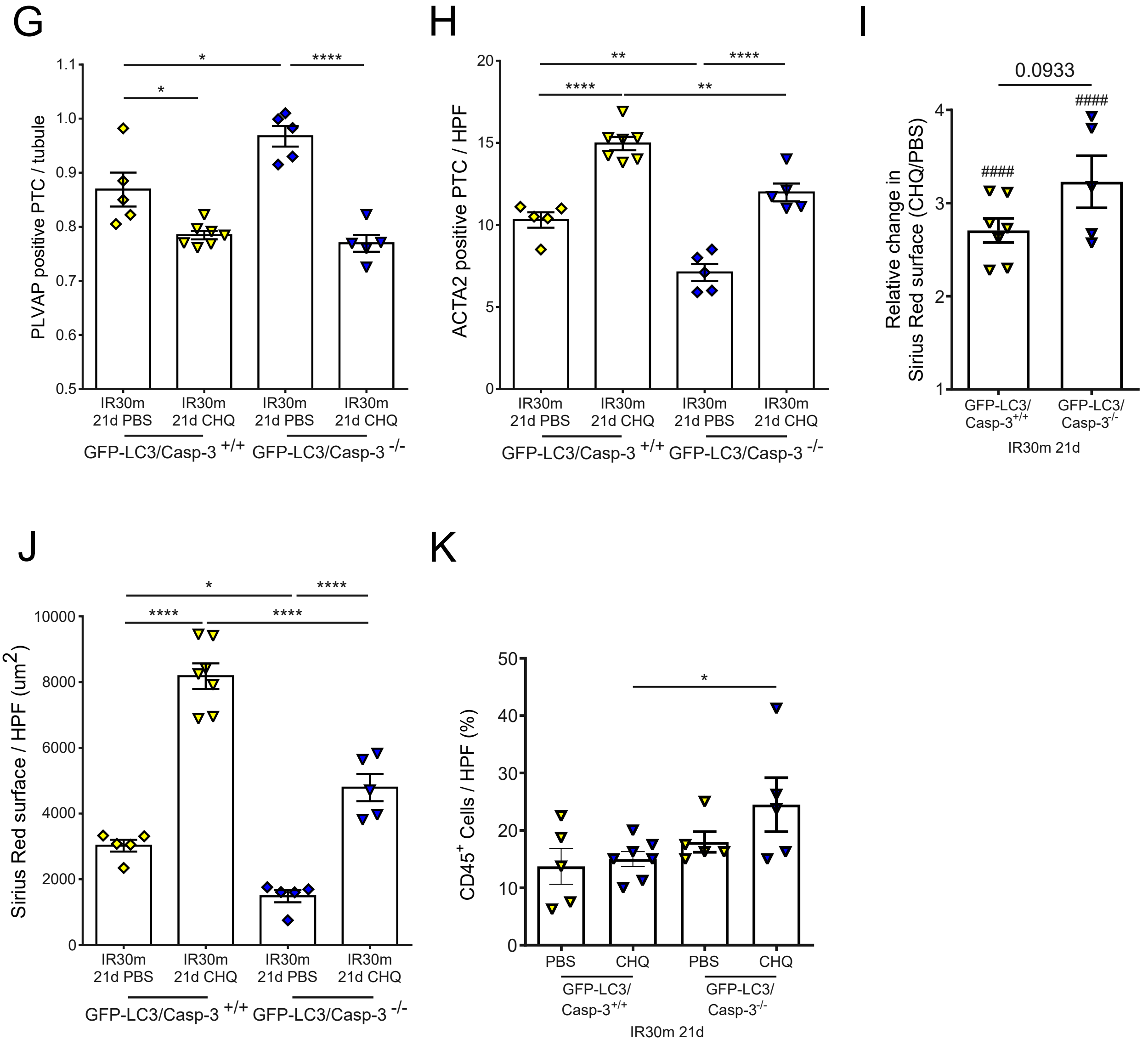
G
PLVAP positive PTC / tubule
1.1
1.0
0.9
0.8
0.7
0.6
0.5
*
*
****
IR30m 21d PBS
IR30m 21d CHQ
IR30m 21d PBS
IR30m 21d CHQ
GFP-LC3/Casp-3 +/+
GFP-LC3/Casp-3 -/-
H
ACTA2 positive PTC / HPF
20
15
10
5
0
**
****
****
**
IR30m 21d PBS
IR30m 21d CHQ
IR30m 21d PBS
IR30m 21d CHQ
GFP-LC3/Casp-3 +/+
GFP-LC3/Casp-3 -/-
I
Relative change in Sirius Red surface (CHQ/PBS)
0.0933
####
####
4
3
2
1
GFP-LC3/ Casp-3+/+
GFP-LC3/ Casp-3-/-
IR30m 21d
J
Sirius Red surface / HPF (um2)
10000
8000
6000
4000
2000
0
*
****
****
****
IR30m 21d PBS
IR30m 21d CHQ
IR30m 21d PBS
IR30m 21d CHQ
GFP-LC3/Casp-3 +/+
GFP-LC3/Casp-3 -/-
K
CD45+ Cells / HPF (%)
50
40
30
20
10
0
*
PBS
CHQ
PBS
CHQ
GFP-LC3/ Casp-3+/+
GFP-LC3/ Casp-3-/-
IR30m 21d

Supplementary References

1. Yang B, Lan S, Dieudé M, et al. Caspase-3 Is a Pivotal Regulator of Microvascular Rarefaction and Renal Fibrosis after Ischemia-Reperfusion Injury. *J Am Soc Nephrol*. Jul 2018;29(7):1900-1916. doi:10.1681/asn.2017050581
