# Supplementary material for: Microvascular autophagy and caspase-3 activation are central regulators of renal fibrosis after ischemia-reperfusion
Source: JCI Insight. 2026 Jul 8;11(13):e201945. doi: 10.1172/jci.insight.201945 (PMC13460856; doi:10.1172/jci.insight.201945)
Supplement: Supplementary file 2 [file jciinsight-11-201945-s093.pdf]

Unedited Western blots for Figure S4A

Colorimetric

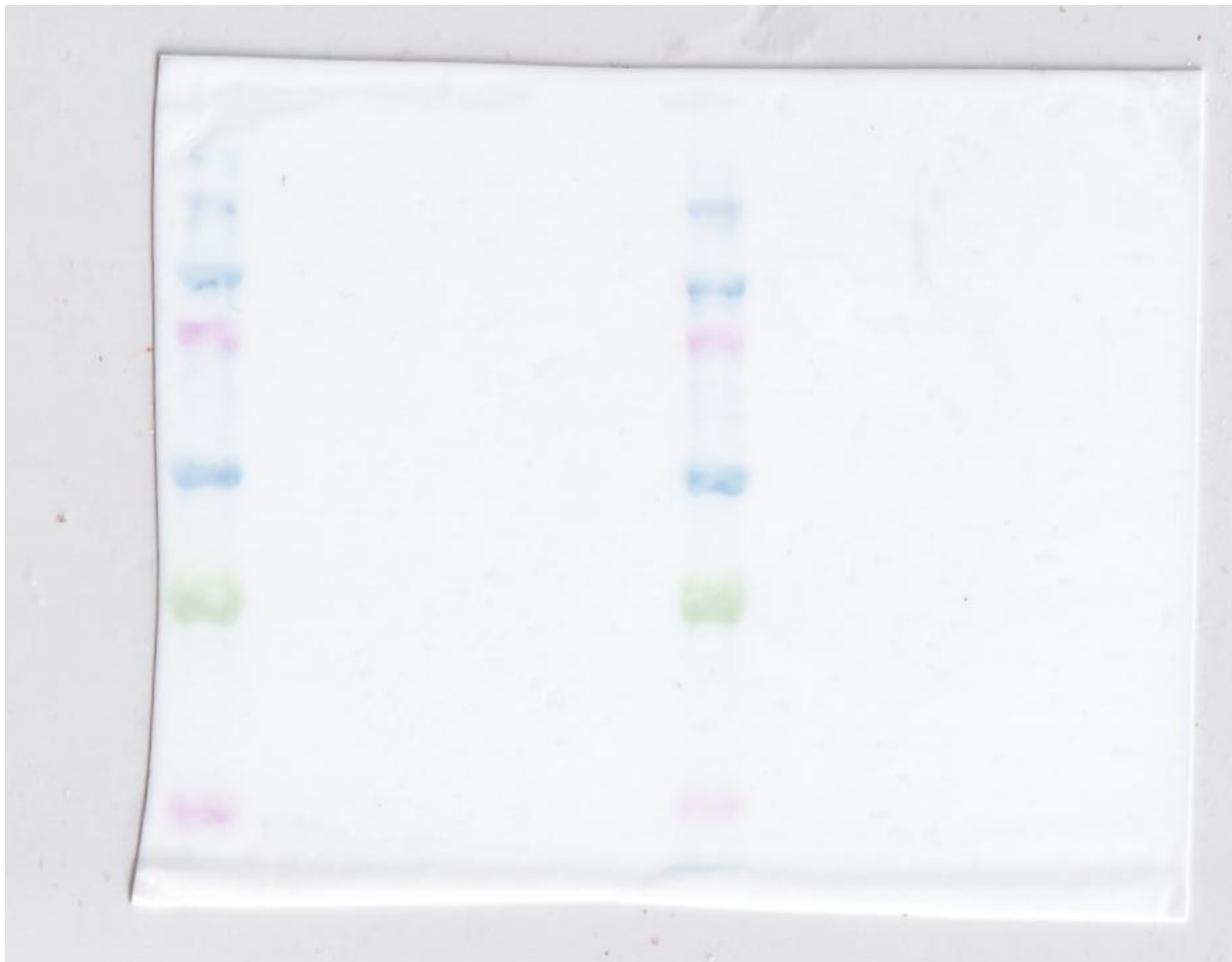

SQSTM1/p62

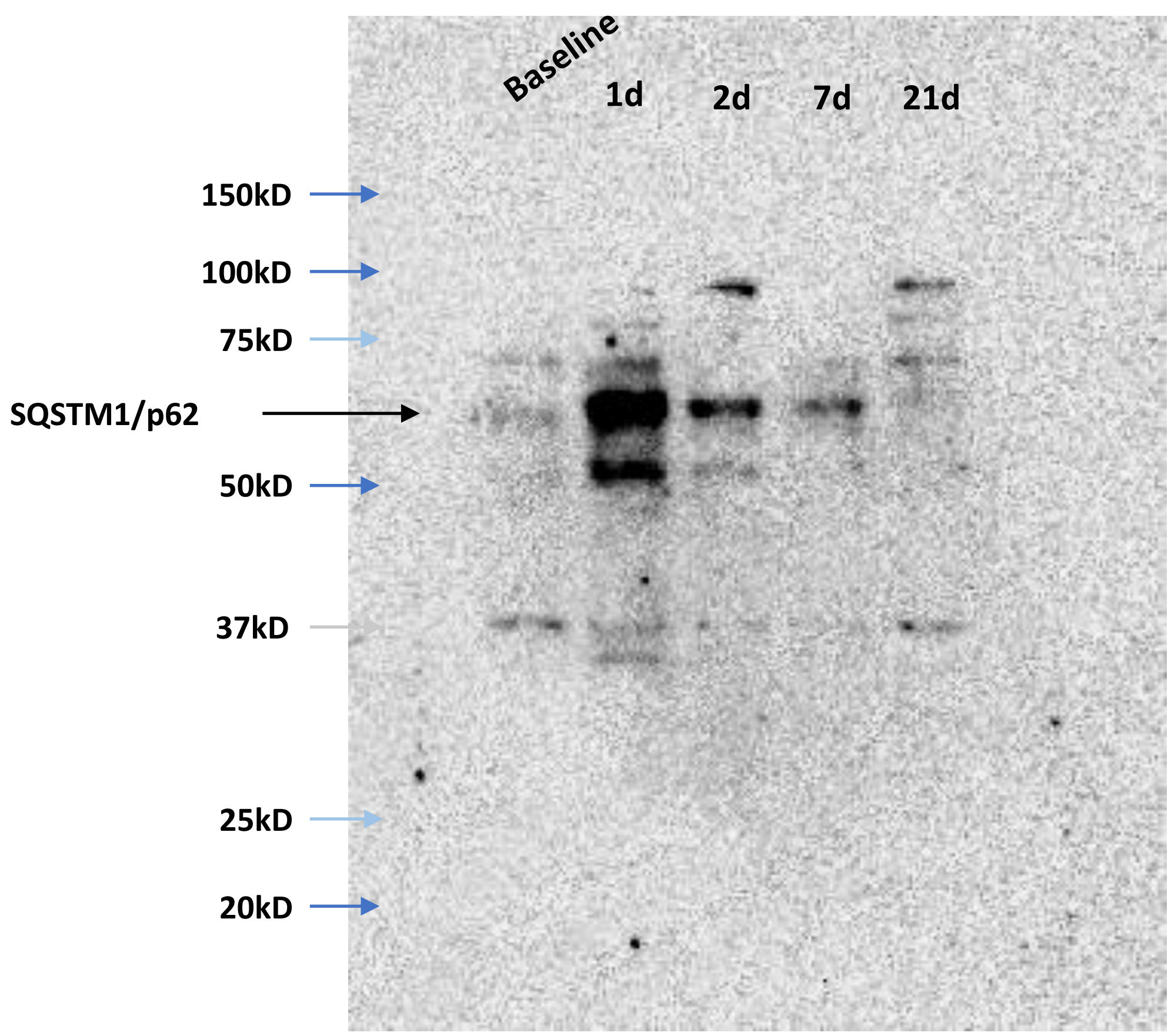

GFP

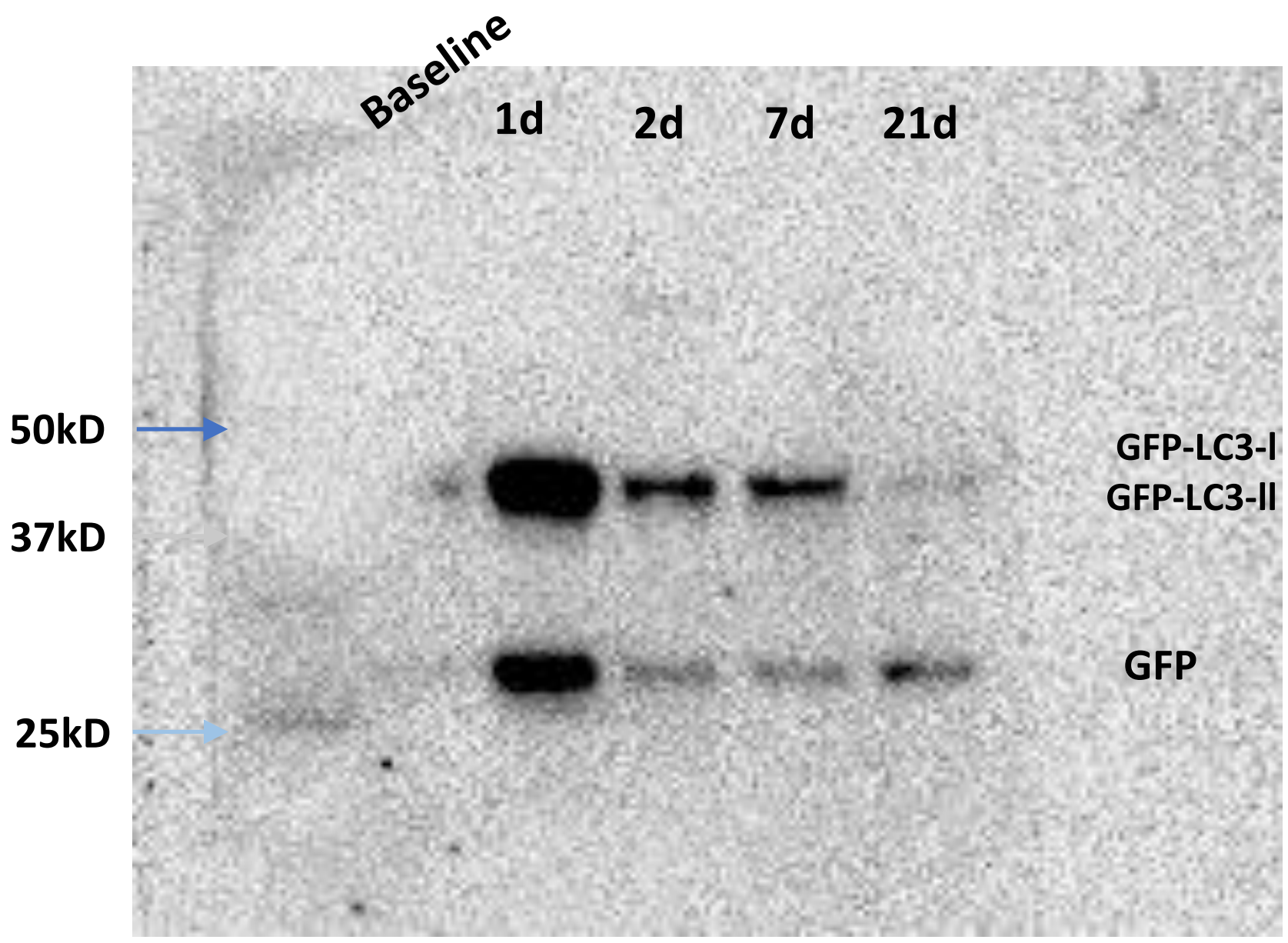
Baseline
1d
2d
7d
21d
50kD
37kD
25kD
GFP-LC3-I
GFP-LC3-II
GFP

LC3-II/I

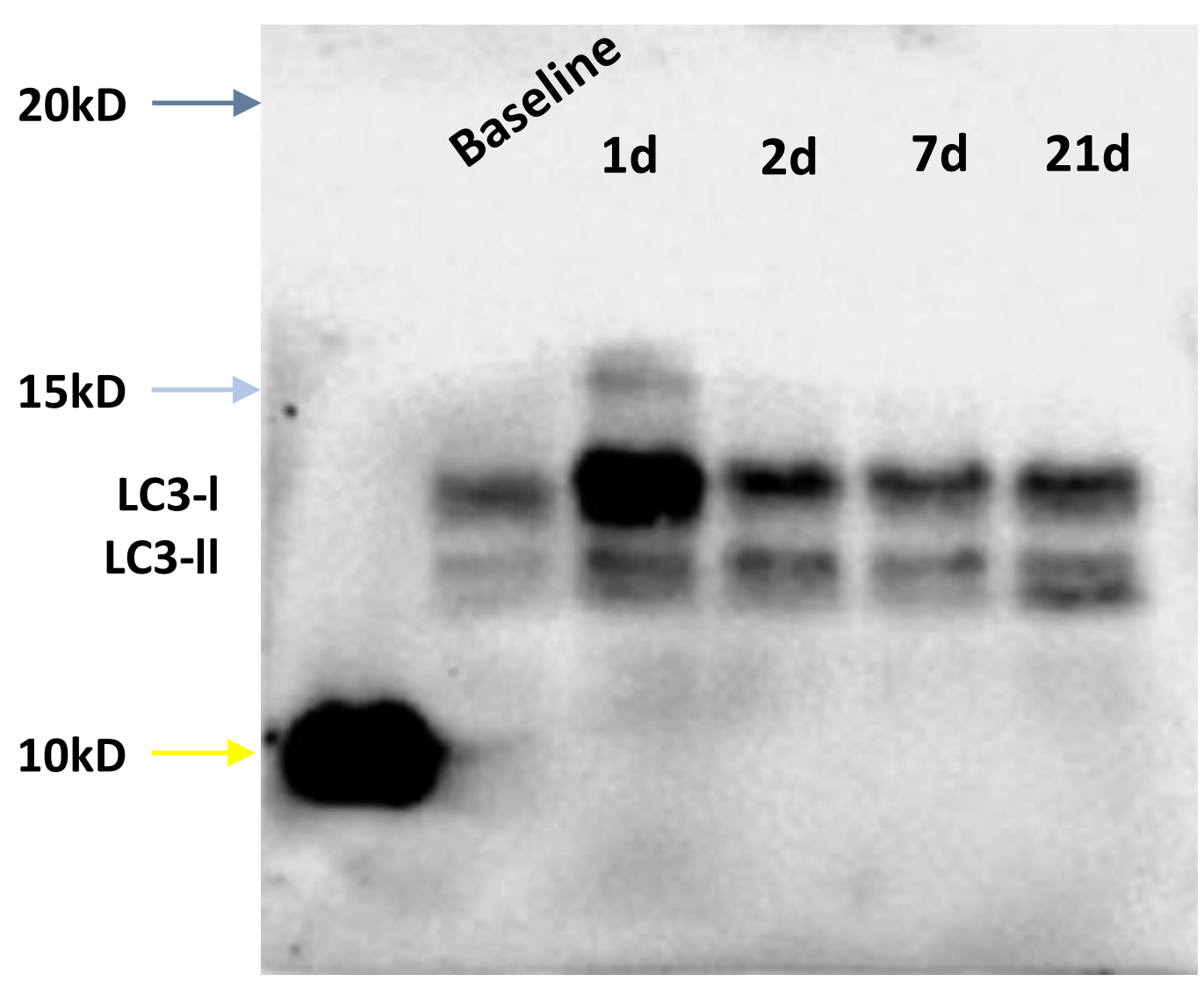
20kD
Baseline
1d
2d
7d
21d
15kD
LC3-I
LC3-II
10kD

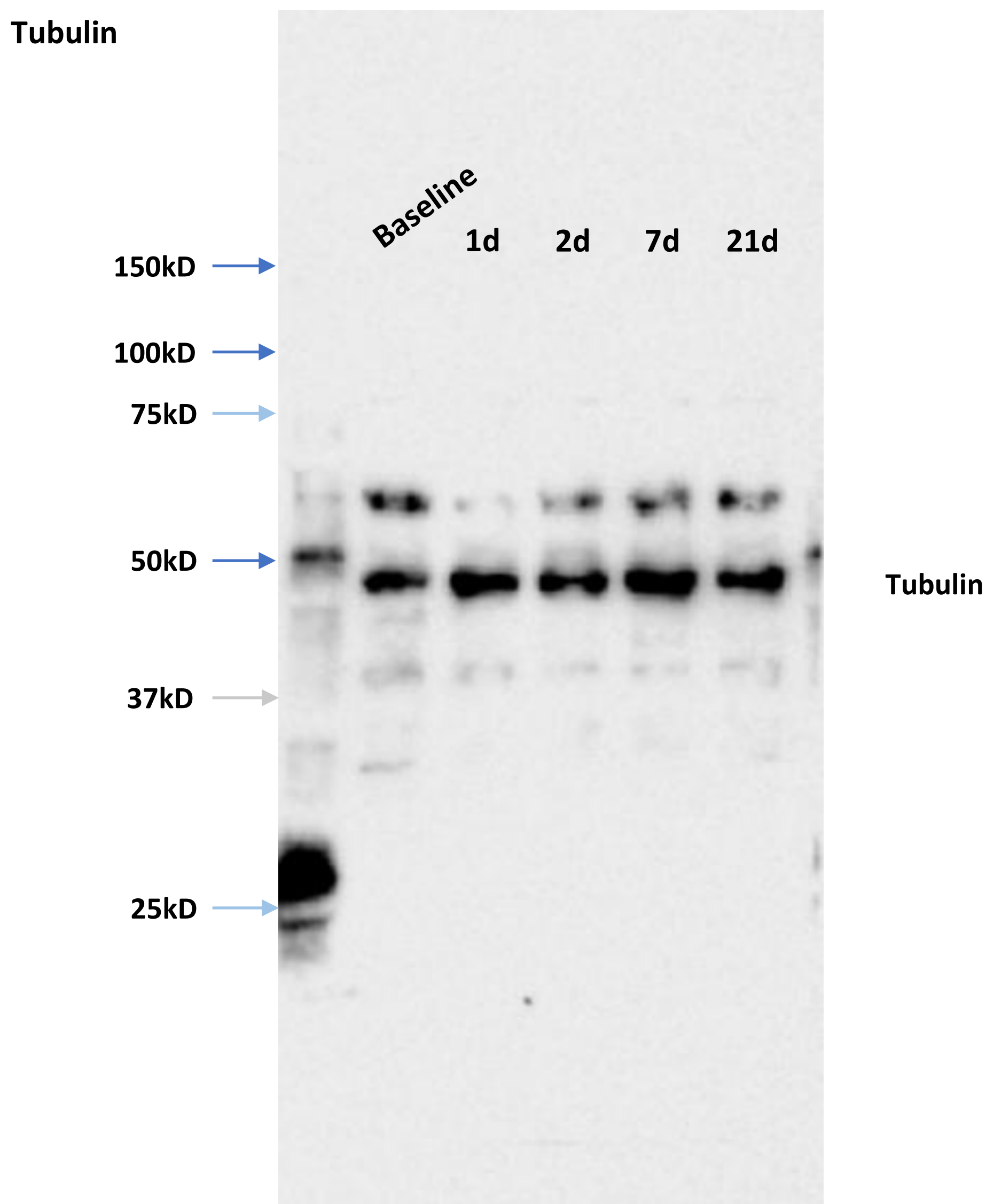
Tubulin
Baseline
1d
2d
7d
21d
150kD
100kD
75kD
50kD
37kD
25kD
Tubulin

Unedited Western blots for Figure S9B

Colorimetric

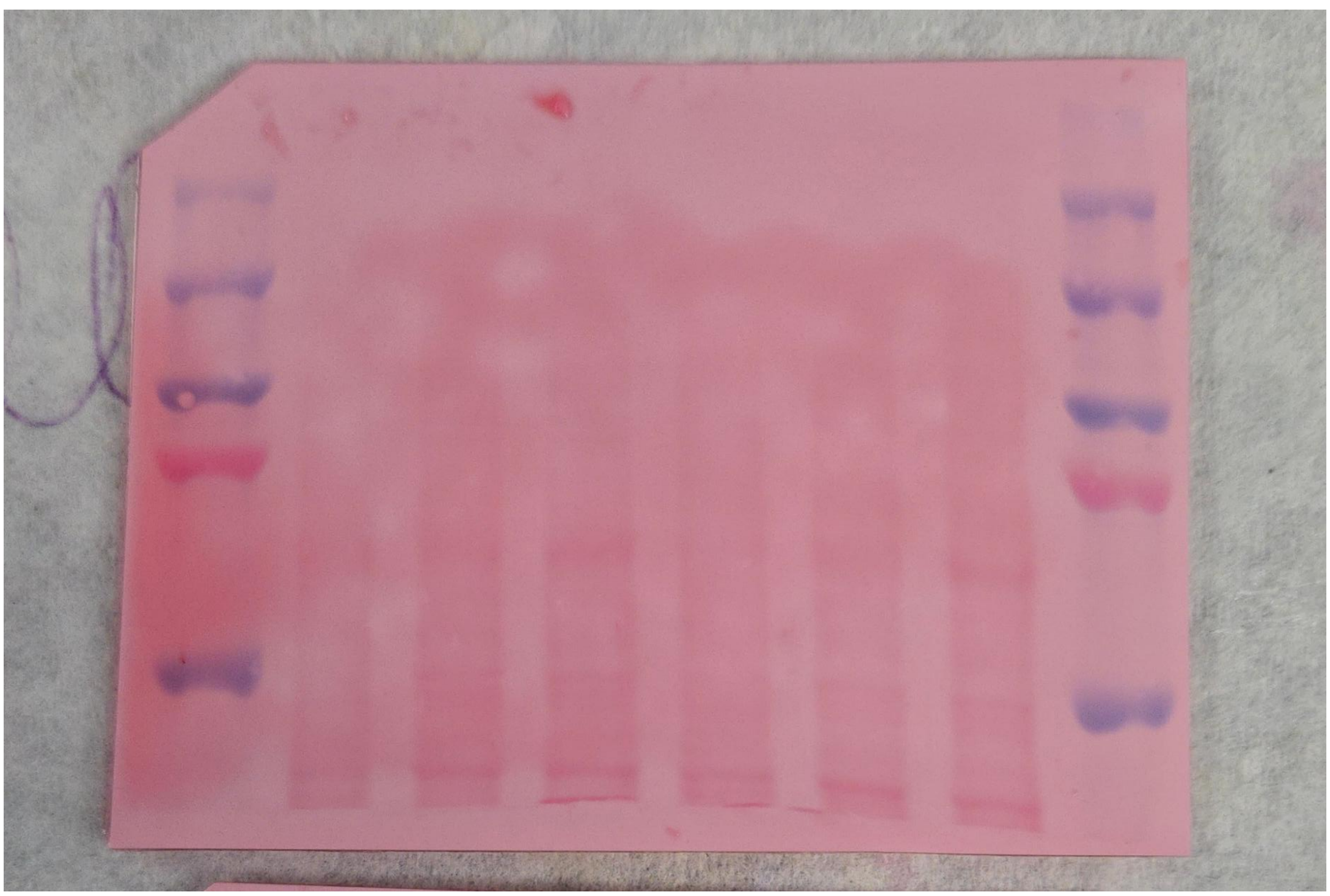

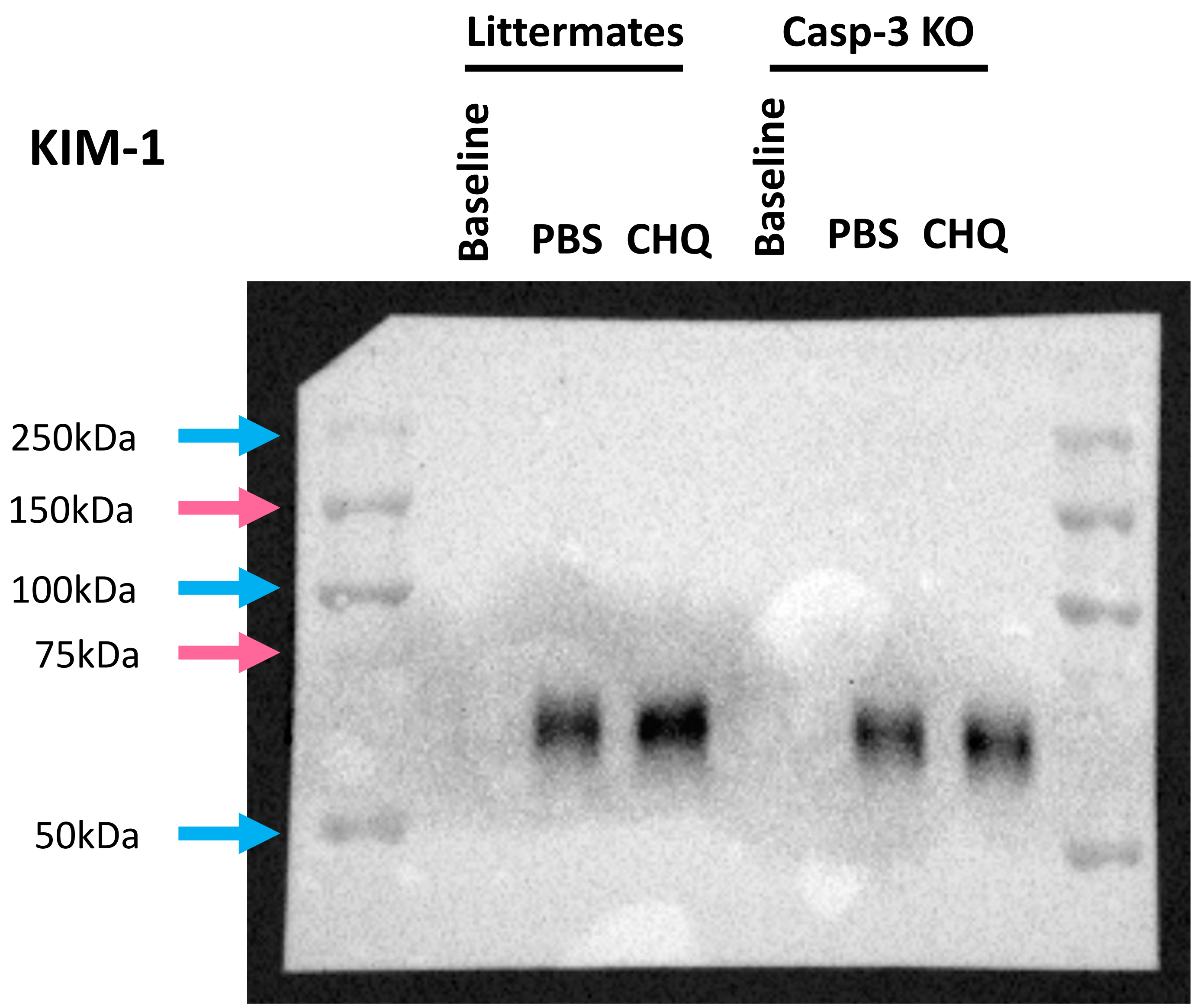
KIM-1
Littermates
Casp-3 KO
Baseline
PBS
CHQ
Baseline
PBS
CHQ
250kDa
150kDa
100kDa
75kDa
50kDa

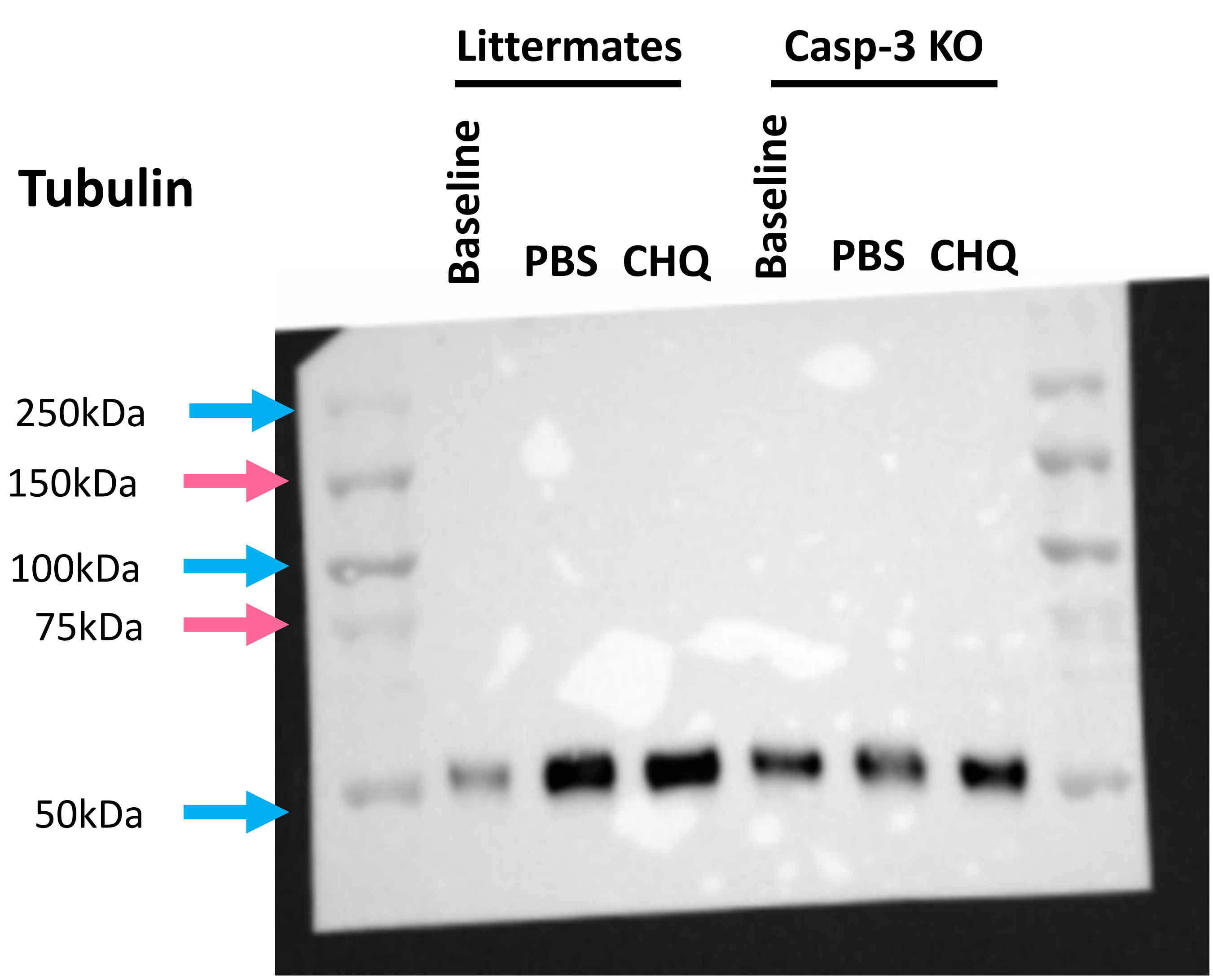
Tubulin
Littermates
Casp-3 KO
Baseline
PBS
CHQ
Baseline
PBS
CHQ
250kDa
150kDa
100kDa
75kDa
50kDa
